# Lack of the Delta Subunit of RNA Polymerase Increases Virulence Related Traits of *Streptococcus mutans*

**DOI:** 10.1371/journal.pone.0020075

**Published:** 2011-05-19

**Authors:** Xiaoli Xue, Helena Sztajer, Nora Buddruhs, Jörn Petersen, Manfred Rohde, Susanne R. Talay, Irene Wagner-Döbler

**Affiliations:** 1 Research Group Microbial Communication, Division of Cell Biology, Helmholtz Centre for Infection Research GmbH, Braunschweig, Germany; 2 Research Group Roseobacter Molecular Systematics, German Collection of Microorganisms and Cell Cultures GmbH, Braunschweig, Germany; 3 Department of Medical Microbiology, Helmholtz Centre for Infection Research GmbH, Braunschweig, Germany; University of California Merced, United States of America

## Abstract

The delta subunit of the RNA polymerase, RpoE, maintains the transcriptional specificity in Gram-positive bacteria. Lack of RpoE results in massive changes in the transcriptome of the human dental caries pathogen *Streptococcus mutans*. In this study, we analyzed traits of the Δ*rpoE* mutant which are important for biofilm formation and interaction with oral microorganisms and human cells and performed a global phenotypic analysis of its physiological functions. The Δ*rpoE* mutant showed higher self-aggregation compared to the wild type and coaggregated with other oral bacteria and *Candida albicans*. It formed a biofilm with a different matrix structure and an altered surface attachment. The amount of the cell surface antigens I/II SpaP and the glucosyltransferase GtfB was reduced. The Δ*rpoE* mutant displayed significantly stronger adhesion to human extracellular matrix components, especially to fibronectin, than the wild type. Its adhesion to human epithelial cells HEp-2 was reduced, probably due to the highly aggregated cell mass. The analysis of 1248 physiological traits using phenotype microarrays showed that the Δ*rpoE* mutant metabolized a wider spectrum of carbon sources than the wild type and had acquired resistance to antibiotics and inhibitory compounds with various modes of action. The reduced antigenicity, increased aggregation, adherence to fibronection, broader substrate spectrum and increased resistance to antibiotics of the Δ*rpoE* mutant reveal the physiological potential of *S. mutans* and show that some of its virulence related traits are increased.

## Introduction

*Streptococcus mutans* is the main causative agent of human dental caries, which is one of the most prevailing infectious diseases in the world [1]. Since *S. mutans* causes damage to the tooth surface of the host, it is considered a cariogenic pathogen [2]. The key factors contributing to the pathogenesis are its strong acidogenicity and aciduricity. *S. mutans* produces acid through metabolism of a wide variety of carbohydrates, which can lower the pH down to pH 4 and causes demineralization of tooth enamel [3], [4]. Moreover, *S. mutans* has a well equipped acid defense system [5], thus it has growth advantages compared to other non-aciduric bacteria. In the acidic stage of the caries process, *Lactobacilli* and *S. mutans* therefore become dominant [6]. Furthermore, to avoid being cleared by saliva and to survive in the highly fluctuating conditions of the oral cavity, it needs to adhere to surfaces, and the best way is the formation of biofilms. *S. mutans* produces extracellular polysaccharides and surface adhesins that mediate its adherence and interaction with other microorganisms in the oral cavity [7], [8]. The polymicrobial community within the biofilms profits from metabolic and genetic communication and is protected against external stress factors [9]. Sophisticated communication mechanisms have been discovered, e.g. *S. mutans* produces, secretes, and senses the peptide pheromone competence-stimulating peptide (CSP) which controls its genetic competence [10], biofilm formation [11], and bacteriocin production [12]. *S. mutans* also interacts with the pathogenic fungus *Candida albicans*, whose hyphae formation is suppressed by CSP [13], as well as by a secreted fatty acid signal, trans-decenoic acid [14].

Another aspect of *S. mutans* pathogenesis is its ability to infect host cells. While *S. mutans* is not as invasive as other streptococci, there are also highly invasive strains/serotypes known [15]. It is frequently detected in heart valve and atheromatous plaque specimens [16], and has been isolated from infective endocarditis [17], suggesting that it is a possible causative agent. The pathogenesis of streptococci [2] includes: (1) adherence to the tissue surface, mainly through binding to the human extracellular matrix (ECM) components, e.g. fibronectin, via surface antigens and other surface structures. Several ECM binding proteins have been identified in *S. mutans* [18]. (2) Survival in the host. For example, a newly isolated *S. mutans* strain was shown to have lower cariogenicity but higher virulence in the blood due to loss of important antigens that allow to escape host phagocytosis [19]. (3) Invasion into and survival within host cells. A serotype f *S. mutans* OMZ175 was capable to invade into and survive in the human coronary artery endothelial cells (HEAEC) [15]. (4) Causing damage to the host by modulating host inflammatory response. *S. mutans* can effectively stimulate inflammatory cytokine production of mononuclear cells [20] and endothelial cells [21].

The delta subunit of the RNA polymerase, RpoE, is conserved in low G+C Gram-positive bacteria (Firmicutes). Biochemical studies proved that RpoE reduces unspecific binding of DNA to RNA polymerase and accelerates the core enzyme recycling, thus it is required for transcriptional specificity [22], [23]. RpoE has been shown to be associated with virulence in *S. agalactiae* [24], [25]. In our previous studies, we characterized the function of RpoE for the first time in *S. mutans*, and showed that loss of RpoE caused massive changes in the transcriptome [26] and proteome (Xue et al. submitted). Here, we studied the functional changes in virulence related traits of the Δ*rpoE* mutant. We analysed the effect of RpoE on self- and co-aggregation, biofilm formation and structure, adherence to ECM, and attachment to and invasion of human epithelial cells. Furthermore, a global phenotypic characterization of the metabolic capabilities of the Δ*rpoE* mutant was performed.

## Methods

### Microbial strains and growth conditions

The Δ*rpoE* mutant of *Streptococcus mutans* was constructed by replacing the coding sequence of the *rpoE* gene with an erythromycin resistance cassette [26]. Genetic complementation of the mutant with the *rpoE* gene in *trans* showed reversal to the wild type phenotype, indicating secondary mutations were not present [26]. All experiments were conducted with fresh glycerol stocks from the same culture of the mutant strain to avoid problems of instability or secondary mutations. *S. mutans* UA159 wild type (ACTT 700610) and the Δ*rpoE* mutant [26] were grown in Todd Hewitt broth (Becton Dickinson, USA) supplemented with 1% yeast extract (THBY) at 37°C aerobically (5% CO_2_ enriched). For biofilm assays, *S. mutans* strains were grown in BM medium [27] containing 0.5% (w/v) sucrose (BMS) under anaerobic conditions (80% N_2_, 10% H_2_, 10% CO_2_). Erythromycin was included where indicated at a final concentration of 10 µg/ml for the Δ*rpoE* strain. For coaggregation, strains *Streptococcus oralis* 7, *S. sanguinis* 22, and *Actinomyces naeslundii* (gifts from Dr. G. Conrads, Germany), and *Candida albicans* DSM 11225 were grown in THBY medium with or without supplementation of 0.5% (w/v) sucrose (THBYS).

### Cultivation of epithelial cells

The human epithelial cell line HEp-2 (ATCC CCL23) (Hela derivative) was cultured in Dulbecco's modified Eagle's medium (DMEM; GibcoBRL, Karlsruhe, Germany) supplemented with 10% fetal calf serum (FCS; GibcoBRL), 5 mM glutamine, penicillin (100 U/ml), and streptomycin (100 mg/ml) as described [28]. Primary human large vascular endothelial cells (HUVEC) isolated from umbilical cord were purchased from PromoCell (Cat. No. 12200, Heidelberg, Germany). Endothelial cells were cultured and propagated with a maximum of eight passages in EGM-2 medium (PromoCell) with 5 mM glutamine, penicillin (100 U/ml), and streptomycin (100 mg/ml) according to the supplier's protocol. For adherence and invasion assays, cells were resuspended and seeded onto coverslips in multiwell plates (Nunc, Roskilde, Denmark) at a concentration of 1×10^5^ cells/ml (500 µl/well) and cultivated for 24 hours to reach confluent monolayers. Cultured cells were maintained in a cell incubator at 37°C in an atmosphere containing enriched 5% CO_2_.

### Self-aggregation assay

Self-aggregation assays were carried out as previously described with minor changes [29]. Briefly, cultures of *S. mutans* strains at stationary growth phase were collected by centrifugation (12,000 rpm, 30 sec), washed twice and resuspended in PBS (pH 7.4) to reach an optical density (OD_600_) of about 0.6 and transferred to cuvettes. Samples were incubated at 37°C for 2 h and the OD_600_ was recorded at different time intervals. Before measurement, samples were equilibrated at room temperature for 5 min. Percent of aggregation was calculated as (OD_600_ at time zero−OD_600_ at time×min)/(OD_600_ at time zero)×100%.

### Coaggregation with oral microorganisms

The coaggregation assay was carried out as described before [30], [31]. The interactions of *S. mutans* wild type and the Δ*rpoE* mutant with the oral microorganisms *S. sanguinis*, *S. oralis*, *A. naeslundii*, and *C. albicans* were investigated. The overnight cultures of these strains were 1∶10 diluted in fresh THBY or THBYS (supplied with 0.5% w/v sucrose) media and cultivated until an OD_600_ of about 0.4 had been reached, corresponding to the logarithmic growth phase. The cells were harvested by centrifugation (5000 rpm, 5 min), washed twice and resuspended in coaggregation buffer (0.1 mM CaCl_2_, 0.1 mM MgCl_2_, 0.15 M NaCl dissolved in 1 mM Tris adjusted to pH 8) to give an OD_600_ of about 0.4. Aliquots of 1 ml from each microbial suspension were combined in a falcon tube and vortexed for 10 sec. Individual bacterial suspensions were used as a control. Coaggregation was scored after 90 min according to Cisar et al. [30] by determining the degree of floc formation by viewing the tubes with the naked eye. The scores ranged from − to ++++, described as follows: −, no evidence of coaggregates in the mixed suspensions; +, finely dispersed coaggregates which did not precipitate; ++, clearly visible coaggregates which did not precipitate immediately; +++, large precipitating coaggregates; and ++++, very large coaggregates that precipitated immediately. Additionally, after 90 min of coaggregation, the turbidity (OD_600_) of the supernatant above the flocs was measured to determine the strength of aggregation quantitatively.

### Biofilm detachment

Bacterial cells from the early stationary phase (OD_600_∼2) were collected by centrifugation (12,000 rpm, 30 sec), washed once and then diluted 1∶100 in BMS medium to an initial OD_600_∼0.02. 48-well polystyrene plates (Nunc, Roskilde, Denmark) were inoculated with 400 µl cell suspension and biofilms were cultured anaerobically for 16 h. The planktonic phase was removed, biofilms were washed once with water, and treated with (1) 1 mg/ml Proteinase K (>30 U/mg dry weight) (Sigma, Germany); (2) 0.2 mg/ml DNase I (>60,000 Dornase unit/mg dry weight) (Calbiochem, Germany); (3) 10 mM sodium *meta*-periodate (Thermo Science, Germany). Control wells were treated with water alone. Plates were incubated at 37°C for 1 h, after then the biofilms were quantified by crystal violet staining as described before [26]. For each experiment, eight biological replicates were performed; the mean value and standard deviation were calculated accordingly. The experiment was repeated four times. The results from one representative experiment are shown.

### Inhibition of biofilm growth

*S. mutans* strains at late exponential phase (OD_600_∼1) were collected by centrifugation (12,000 rpm, 30 sec), washed once and resuspended in BMS medium supplied with 1 mg/ml Proteinase K, while control cells were grown in BMS medium alone. Biofilms were cultured in 48-well polystyrene plates for 1, 2, 3, 4 h, and after then the biofilms were quantified by crystal violet staining as described above. For each experiment, eight biological replicates were performed; the mean value and standard deviation were calculated accordingly. The experiment was repeated four times. The results from one representative experiment are shown.

### Analysis of the extracellular biofilm matrix

Bacterial cells of *S. mutans* strains from the early stationary phase (OD_600_∼2) were collected by centrifugation (12,000 rpm, 30 sec), washed once and then diluted 1∶100 in BMS medium to an initial OD_600_∼0.02. Biofilms were grown in polystyrene petri dishes (Nunc, Roskilde, Denmark) in 5 ml BMS medium for 16 h anaerobically. The planktonic phase was removed, and the biofilms were washed three times with sterile water to remove loosely bound material. The biofilm cells were collected by scraping and resuspended in sterilized water supplemented with a protease inhibitor cocktail (Cat. No. 04693124001, Roche, Germany) and chloramphenicol (50 µg/ml). For each strain, eight biofilm samples were pooled (final volume of 5 ml) and digested with 75 U/ml N-glycanase (NEB, England) at 37°C for 1 h to disrupt the biofilm flocs. The digested samples were used for biomass, polysaccharide, extracellular DNA and protein determinations. For biomass (dry weight) determination, three volumes of cold ethanol (−20°C) were added to 1 volume of biofilm suspension, and centrifuged at 10,000 g for 5 min at 4°C. The pellets were washed two times with cold ethanol, vacuum dried (Eppendorf Concentrator 5301, Germany) and weighed. The extracellular polysaccharides (soluble and insoluble) were extracted as described before [32]. The amount of total carbohydrates was determined by the phenol-sulfuric acid method [33]. Extracellular DNA was extracted using the cetyltrimethylammonium bromide (CTAB)-DNA precipitation method [34], [35]. For extracellular protein extraction, 500 µl of (1) 0.5 M NaOH containing 5 mM EDTA [36]; or (2) 0.5% Triton X-100 were added to 2 ml biofilm samples and incubated at 4°C for 1 h with agitation. The extracts were centrifuged at 10,000 g for 10 min at 4°C. One volume of a solution containing 20% trichloracetic acid (TCA), 80% acetone and 0.14% 2-mercaptoethanol (2ME) was added to the supernatant and proteins were precipitated at −20°C for at least 1 h. Pellets were collected by centrifugation (10,000 g for 10 min), washed with acetone supplemented with 0.07% 2ME, and kept at −20°C for at least 1 h. Pellets were collected by centrifugation (10,000 g for 15 min) and vacuum dried (Eppendorf Concentrator 5301, Germany). Pellets were resuspended in 150 µl 50 mM Tris buffer (pH 6.8). The protein concentration was determined using the Coomassie (Bradford) protein assay kit (Thermo Science, Germany). The extracellular proteins extracted from about 5 mg biomass of the wild type and the Δ*rpoE* mutant were subjected to SDS-PAGE using 7.5% and 12% separating gels. The gels were stained using EZBlue Gel staining reagent (Sigma, Germany) according to the manufacturer's instruction. Prestained protein standard marker (BioRad, Germany) was used. The interesting protein bands were excised, digested with trypsin, and analyzed by MALDI-TOF MS (Matrix-Assisted-Laser-Desorption/Ionization-Time-Of-Flight Mass Spectrometry) as described before [37]. The obtained peptide masses were used for protein identification by peptide mass fingerprinting (PMF) using the MASCOT program. Protein score is −10*Log(P), where P is the probability of a random event; scores higher than 81 are considered as significant (P<0.05). For proteins not identified by MALDI-TOF/PMF, the most abundant peptide ions are then subjected to MS/MS analysis to determine the sequence. The results from both analyses were combined and searched for protein identification using the program MASCOT. Ions score is −10*Log(P), while P is the probability of a random event. Ions scores >51 indicate identical peptide identity or extensive homology (P<0.05). Protein scores are then derived from ions scores as a non-probabilistic basis.

### Binding to human extracellular matrix (ECM) components

ECM microtiter plates precoated with collagen I, collagen II, collagen IV, fibronectin, laminin, tenascin and vitronectin and BSA (as a negative control) were purchased from Chemicon (Millipore, Germany). The ECM assay was performed as described before with minor modifications [38]. Briefly, the overnight cultures of *S. mutans* wild type and the Δ*rpoE* mutant were 1∶10 diluted with fresh THBY medium and cultivated until late exponential phase (OD_600_∼1.0). 100 µL of cultures were inoculated into precoated wells and incubated for 2 h at 37°C without agitation. In parallel, bacterial cultures were serially diluted and plated on THBY agar plates in three replicates for colony forming unit (CFU) counting. After 2 h incubation, the ECM plate was washed two times with PBS (pH 7.4) and dried for 20 min under the cleanbench. The attached bacteria were stained with 100 µl of 0.4% crystal violet at room temperature for 45 min. After washing five times with PBS and drying under the cleanbench, the crystal violet dye was extracted by adding 100 µL of absolute ethanol and incubating for 2 h at room temperature with agitation. The supernatant (50 µl) was transferred to a microtiter plate and absorbance was measured at 620 nm with the Wallac Victor multi-label counter (Perkin-Elmer, Germany). For each ECM component, both strains were tested in five replicates, while the THBY medium without cells was measured in two replicates as a negative control. Mean and standard deviation were calculated from the replicate samples.

### Bacterial adherence and invasion assays

Monolayers of epithelial or endothelial cell were cultivated as described above. The media were exchanged to fresh media (without antibiotics), and the cells were incubated for 1 h. *S. mutans* wild type and the Δ*rpoE* mutant from the stationary growth phase (OD_600_∼2) were harvested by centrifugation (5000 rpm, 20 min), washed once with PBS and adjusted to an OD_600_∼1, and diluted 1∶20 in DMEM (HEp-2 medium) or EGM (HUVEC medium) supplied with 5% fetal calf serum. Bacterial density was determined by CFU counting. 250 µl bacterial inoculum (5×10^7^ CFU/ml) were added to the epithelial or endothelial cells at a multiplicity of infection (MOI) of 125∶1 (1.25×10^7^ CFU bacteria at 1×10^5^ cells per well), and incubated for 1–4 hours. The supernatant was taken for quantification of the lactate dehydrogenase (LDH) enzyme that is released upon cell lysis using the CytoTox 96 non-radioactive cytotoxicity assay (Promega, Germany) according to the manufacturer's instruction.

### Immunofluorescence microscopy

Epithelial or endothelial cells infected with *S. mutans* strains were washed twice with fresh medium, fixed with PBS containing 4% paraformaldehyde, and stained for extra- and intracellular bacteria using a polyclonal rabbit anti-*S. mutans* antibody (Abcam, USA) and Alexa Fluor 488-(green) and 568-(red) conjugated goat anti-rabbit IgG as described before [39]. Immunofluorescence was visualized under the Axiophot microscope (Zeiss) and eight to ten random images of each coverslip were recorded using the AxioCam HRc camera and AxioVision software (version 4.7). The number of cells and adherent bacteria were counted. For each image, the mean value and standard deviation of the ratio of bacteria/cell were calculated from six coverslips obtained from three independent experiments.

### Field emission scanning electron microscopy (FESEM)

HEp-2 cells infected with *S. mutans* strains were fixed with 5% formaldehyde and 2% glutaraldehyde in cacodylate buffer (0.1 M cacodylate, 0.01 M CaCl_2_, 0.01 M MgCl_2_, 0.09 M sucrose, pH 6.9). For morphological analysis of the biofilm structure, biofilms of *S. mutans* wild type and the Δ*rpoE* mutant were grown on plastic coverslips as described above for the determination of extracellular biofilm matrix components. Biofilms were washed and fixed with 5% formaldehyde and 2% glutaraldehyde in cacodylate buffer, dehydrated, critical-point dried and sputter coated with gold as described before [40]. The images were taken by a field emission scanning electron microscope (Zeiss DSM 982 Gemini) using the Everhart Thornley SE detector and the inlens detector in a 50∶50 ratio at an acceleration voltage of 5 kV.

### Phenotype microarray (PM) tests

PM tests were performed using Biolog's PM facility of the DSMZ, essentially as described elsewhere [41], [42]. The PM experiments included eight metabolic arrays supplied with different carbon sources, nitrogen sources, phosphorus sources, and sulphur sources (PM 1–8) and twelve sensitivity arrays that contained inhibitory compounds (PM 9–20). Both the wild type and the Δ*rpoE* mutant were subjected to full 20-panel PM analysis according to the Biolog's procedures for “*Enterococcus faecalis* and other lactic acid bacteria” (Supporting Information S1). To improve the response of *S. mutans* for PM 3–PM 8, four different pH values (5.5 to 7.0) for tricarballylic acid pH were tested in a pilot experiment with PM 3. The best results could be obtained at pH 6.5, and this pH was used in the subsequent experiments for PM 3–PM 8.The PM tests were repeated in two independent experiments. The formation of the tetrazolium redox dye was recorded every 15 min for 96 hours, and data were analyzed with the OmniLog-File Management and Parametric Management softwares (version 1.20.02). A height difference threshold of 50 for metabolic arrays (PM 1–PM 8) and a threshold of 100 for sensitivity assays (PM 9–PM 20) was used to determine metabolic activity differences. Detailed information about the PM technique is available at http://www.biolog.com.

### Microarray Data

All microarray data discussed in this paper is MIAME compliant as detailed on the MGED Society website http://www.mged.org/Workgroups/MIAME/miame.html and has been deposited in a MIAME compliant database (GEO accession no. GSE22333). All the methods related to the microarray experiment (experimental set up, microarray design, RNA extraction, labeling, hybridization, scanning, and the computational methods used for data analysis) have been published previously [26].

## Results and Discussion

### Increased self- and co-aggregation in the Δ*rpoE* mutant

**Figure 1:**
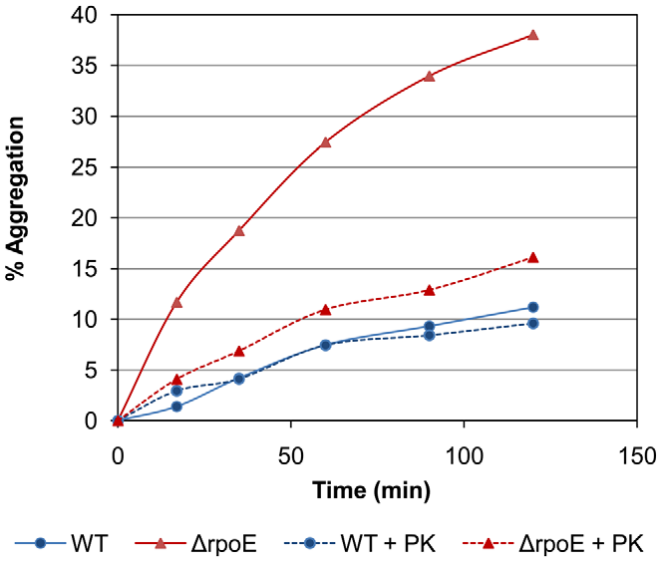
Self-aggregation of *S. mutans* wild type (▪) and the Δ*rpoE* mutant (□). Bacterial cells were in PBS buffer without (solid lines) and with Proteinase K treatment (dashed lines). Percent of aggregation was calculated as (OD_600_ at time zero−OD_600_ at time×min)/(OD_600_ at time zero)×100%. The representative results from three independent experiments are shown. The Δ*rpoE* mutant showed higher self-aggregation compared to the wild type. Pre-treatment with Proteinase K diminished the self-aggregation of the Δ*rpoE* mutant.

The *S. mutans* cells from the stationary growth phase were used for the self-aggregation analysis. The Δ*rpoE* mutant showed higher self-aggregation than the wild type in PBS buffer (Fig. 1). The reduction in optical density (OD_600_) in the Δ*rpoE* mutant reached 38% after 2 hours, while the wild type obtained only 11% reduction. Proteinase K treatment had no obvious effect on the wild type under these conditions. By contrast, proteinase K treatment strongly reduced the self-aggregation of the Δ*rpoE* mutant, suggesting that surface proteins contribute to its self-aggregation. However, the self-aggregation of the Proteinase K treated Δ*rpoE* mutant was still higher than that of the wild type, thus a different surface structure would be expected in the mutant.

Dental biofilms start with the adherence of the initial colonizers, e.g. Streptococci, and *Actinomyces* [43], to the exposed salivary pellicle. Then genetically distinct species of microorganisms coaggregate with these pioneer colonizers through specific receptors [44]. The ability of *S. mutans* to coaggregate with other oral microorganisms, e.g. the initial colonizers *S. oralis*, *S. sanguinis*, and *A. naeslundii* was therefore investigated, as well as the coaggregation with the yeast *C. albicans*. Following the procedure described by Periasamy et al. [31], cells grown in THBY medium until the exponential phase were collected and transferred into the coaggregation buffer to give a similar cell density (OD_600_ about 0.4) before each pair of two strains was mixed together. The result was recorded after 90 min of coaggregation, and the self-aggregation of each strain was used as a control. As shown in table 1A, the wild type had weak self- and co-aggregation, and no visible flocs were formed. By contrast, the Δ*rpoE* mutant formed flocs in the self-aggregation test and coaggregated with *S. sanguinis*, *A. naeslundii*, and *C. albicans*. This is in line with previous findings that *S. mutans* had weak coaggregation with *A. naeslundii* [45], [46], and with *C. albicans* [47]. The secreted or surface associated proteins in *S. mutans* strains contribute to the bacterial cell-cell interaction, e.g. the surface antigen SpaP [48], have been reported. As shown in table 1B, treatment of Proteinase K reduced the coaggregation capability of both strains, which was indicated by the increased optical density (OD_600_) in the supernatant of Proteinase K treated samples. In addition, the flocs formation of the Δ*rpoE* mutant was eliminated by Proteinase K (table 1B). Expression of the aggregation-mediating proteins depends on the growth phase and the extent of aggregation is also influenced by the buffer, since Proteinase K treatment had no obvious effect on the self-aggregation of the wild type for stationary phase cells suspended in PBS buffer (Fig. 1).

**Table 1:** Coaggregation of *S. mutans* wild type (WT) and Δ*rpoE* mutant with oral microorganisms without (A)/with (B) Proteinase K treatment.

A												
	*S. mutans* WT		*S. mutans* Δ*rpoE*		*C. albicans*		*S. sanguinis*		*S. oralis*		*A. neaslundii*	
*S. mutans* WT	−a	0.23	+	0.14	−	0.10	−	0.24	−	0.24	−	0.23
*S. mutans* Δ*rpoE*			++	0.08	+	0.07	+	0.17	nd	nd	+	0.12
*C. albicans*					−	0.08	−	0.20	−	0.24	−	0.14
*S. sanguinis*							−	0.29	−	0.30	−	0.22
*S. oralis*									−	0.30	−	0.22
*A. neaslundii*											−	0.20

B												
	*S. mutans* WT		*S. mutans* Δ*rpoE*		*C. albicans*		*S. sanguinis*		*S. oralis*		*A. neaslundii*	
*S. mutans* WT	−	0.39	−	0.39	−	0.27	−	0.37	−	0.41	−	0.34
*S. mutans* Δ*rpoE*			−	0.39	−	0.37	−	0.40	−	0.41	−	0.32
*C. albicans*					−	0.14	−	0.27	−	0.28	−	0.20
*S. sanguinis*							−	0.37	−	0.41	−	0.30
*S. oralis*									−	0.42	−	0.34
*A. neaslundii*											−	0.24

a The settlement of flocs is record after 90 minutes as shown in the first column, the number of ‘+’ indicates the strength of floc settlement, while ‘−’ indicates no visible coaggregation flocs, and ‘nd’ means not determined. The optical density (OD_600_) of the supernatant is also recorded as shown in the second column. Since each strain had a similar optical density before mixing, the turbidity changes helped to judge coaggregation strength.

*S. mutans* is known to metabolize sucrose and to produce polysaccharides (glucans and fructans) to promote adherence and aggregation [7]. In agreement with this, supplementation of the THBY growth medium with sucrose resulted in very effective coaggregation for both the wild type and the Δ*rpoE* mutant with all other microorganisms (table 2A), and Proteinase K only partially diminished the coaggregation effect (table 2B), suggesting not only proteins, but also polysaccharides are involved in the coaggregation reaction, which is in line with previous findings [44].

**Table 2:** Coaggregation of sucrose grown *S. mutans* wild type (WT) and Δ*rpoE* mutant with oral microorganisms without (A)/with (B) Proteinase K treatment.

A												
	*S. mutans* WT		*S. mutans* Δ*rpoE*		*C. albicans*		*S. sanguinis*		*S. oralis*		*A. neaslundii*	
*S. mutans* WT	++++	0.01	++++	0.01	+++	0.05	++	0.13	+++	0.09	++	0.15
*S. mutans* Δ*rpoE*			++++	0.01	+++	0.08	++	0.13	+++	0.11	+	0.19
*C. albicans*					−	0.23	+	0.19	+	0.15	−	0.15
*S. sanguinis*							−	0.32	+	0.20	+/−	0.27
*S. oralis*									+	0.18	+	0.24
*A. neaslundii*											+/−	0.28

B												
	*S. mutans* WT		*S. mutans* Δ*rpoE*		*C. albicans*		*S. sanguinis*		*S. oralis*		*A. neaslundii*	
*S. mutans* WT	+++	0.04	++	0.06	−	0.13	++	0.22	++	0.11	++	0.22
*S. mutans* Δ*rpoE*			++	0.08	−	0.17	++	0.29	++	0.13	+	0.49
*C. albicans*					−	0.28	−	0.32	+	0.23	−	0.31
*S. sanguinis*							+	0.39	++	0.18	+/−	0.38
*S. oralis*									++	0.28	+	0.28
*A. neaslundii*											−	0.39

a Same as in table 1.

Although *S. mutans* has multiple mechanisms to bind directly to the tooth pellicle, it is not an initial colonizer [49] and needs to coaggregate with the pioneer species to adhere and build a spatially organized community in dental biofilms. The ability to self-aggregate allows the quicker accumulation of bacterial cells. Therefore, the higher self- and co-aggregation of the Δ*rpoE* mutant may help to become dominant in the oral bacterial community.

### Biofilm structure and biofilm matrix assay

**Figure 2:**
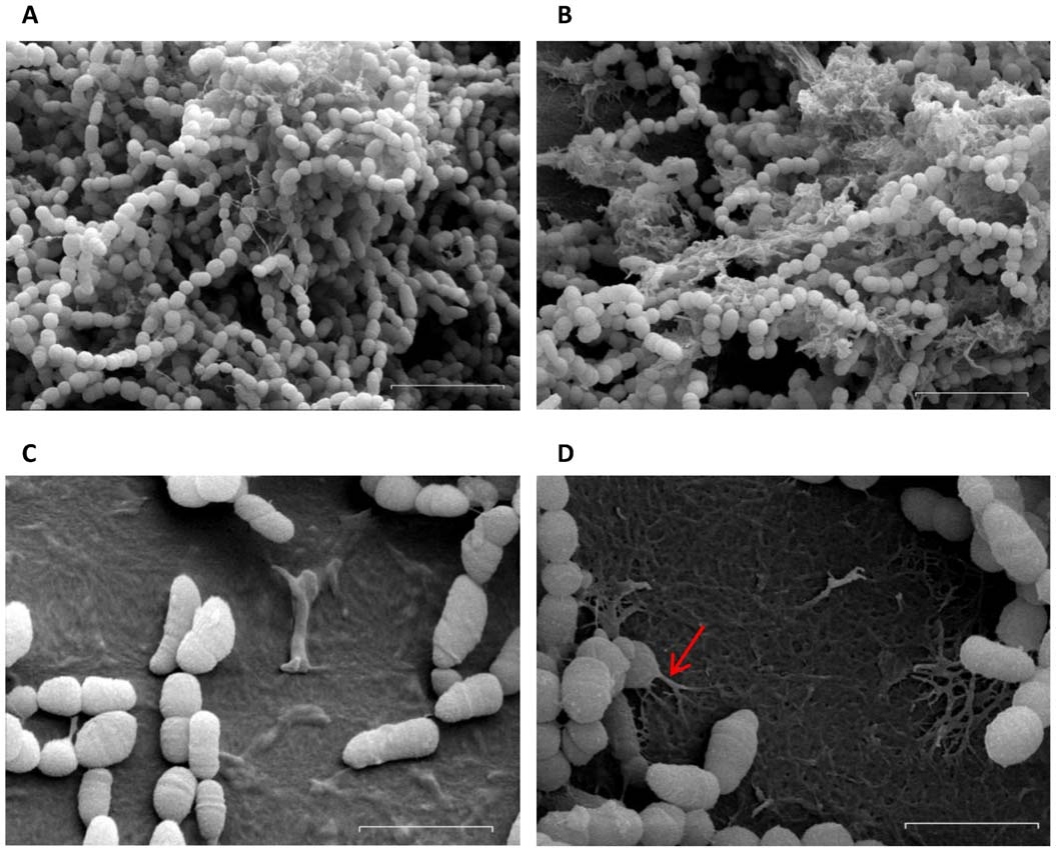
Scanning electron microscopy of 16 h old biofilms of *S. mutans* strains. Wild type (A, C); Δ*rpoE* mutant (B, D). The Δ*rpoE* mutant had a different structure of the biofilm matrix compared to the wild type. The red arrow shows the dendrite-like structure of the Δ*rpoE* mutant biofilm that attached to the polystyrene surface. The bars in image A, B indicate 5 µm, while in image C, D they indicate 2 µm.

Differences in the structure of the biofilm surrounding matrix were revealed under the scanning electron microscope. The wild type biofilm matrix appeared to be more compact (Fig. 2A) than that of the Δ*rpoE* mutant (Fig. 2B). Moreover, unlike the smooth surface formed by the wild type (Fig. 2C), the Δ*rpoE* mutant produced dendrite-like extracellular components to attach on the surface (Fig. 2D, arrow). This is consistent with our previous reports that the Δ*rpoE* mutant formed a clumping inhomogeneous biofilm compared to the wild type [26].

**Figure 3:**
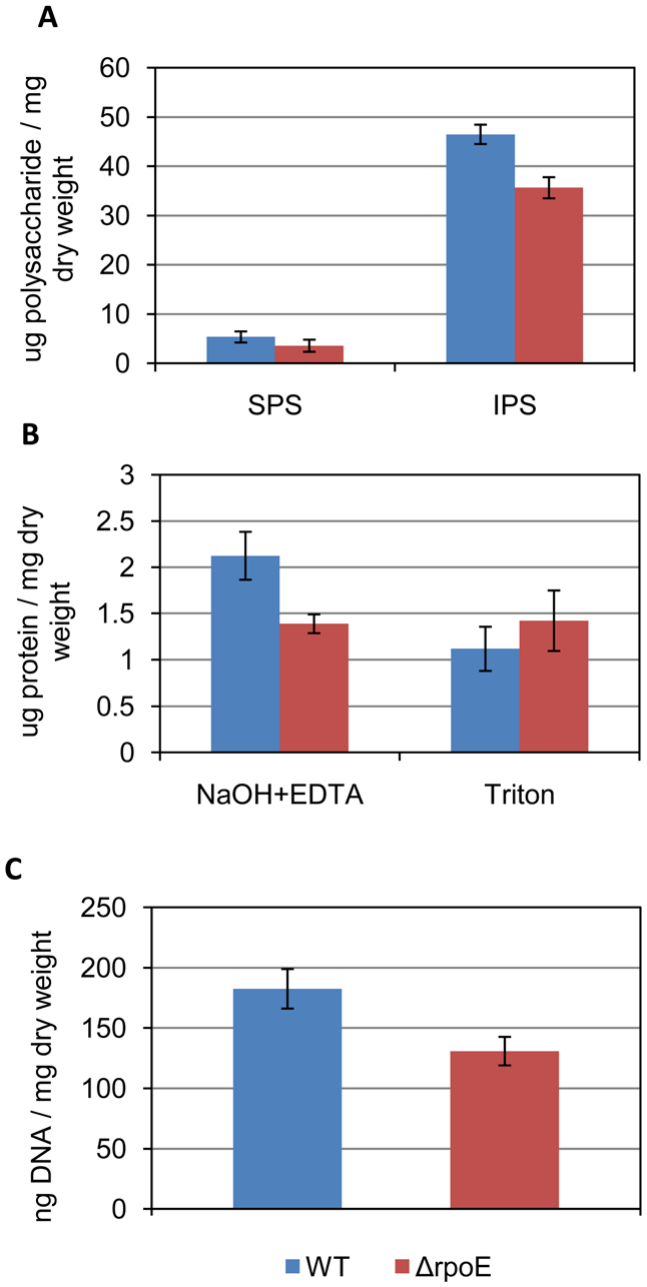
Quantification of extracellular polysaccharides (A), proteins (B), and DNA (C) in *S. mutans* biofilms. Blue columns: wild type; red columns: Δ*rpoE* mutant. Two different methods were used for the extraction of proteins, including EDTA/NaOH and triton extraction. Mean value and standard deviation were calculated from three biological replicates.

The biofilm matrix is comprised of polysaccharides, proteins, and DNA together with other substances [50], thus the total amount of these three major components was quantified. The amount of extracellular insoluble polysaccharides and DNA was slightly less in the Δ*rpoE* mutant than in the wild type when normalized to dry weight (Fig. 3A and C). Lower yield of extracellular proteins was obtained in the Δ*rpoE* mutant using the NaOH/EDTA extraction method, however, a similar amount of proteins was found in both strains by mild detergent triton extraction. Thus the Δ*rpoE* mutant probably had a different extracellular protein composition compared to the wild type (Fig. 3B). To investigate the effect of these components on biofilm formation, sodium *meta*-periodate (NaIO_4_), Proteinase K, and DNase I were used as inhibitors for polysaccharides, proteins, and DNA, respectively. Treatment with Proteinase K caused partial detachment of 16 hour old biofilms of both strains, and moreover, adding the Proteinase K directly to the medium from the beginning of bacterial growth, strongly inhibited the adherence of both strains, thus no biofilm was formed (Figure S1). Thus, the extracellular proteins are necessary for initial adhesion of *S. mutans* to the surface.

**Figure 4:**
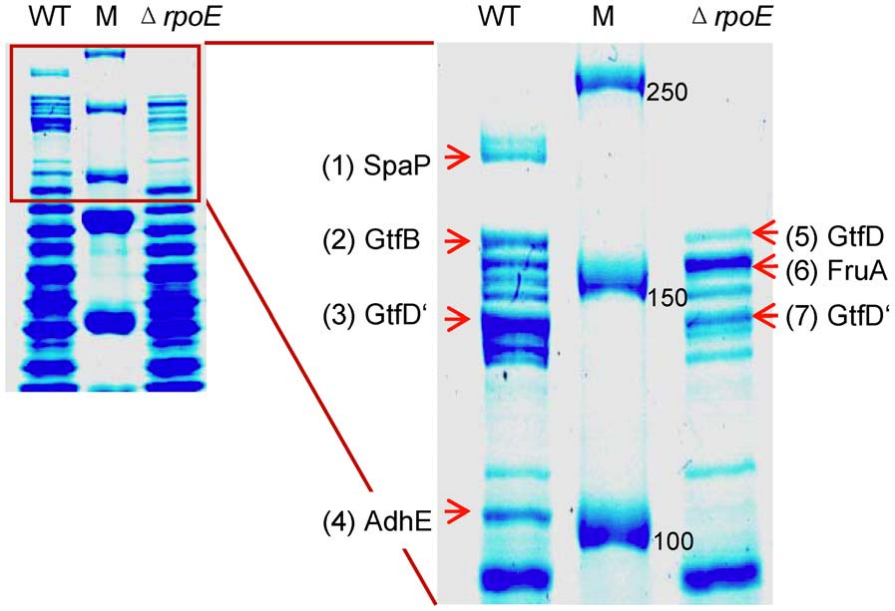
Extracellular matrix proteins of *S. mutans* biofilms. Similar protein amounts extracted from 16 h old biofilms of the wild type (WT) and the Δ*rpoE* mutant were subjected to the 7.5% SDS-PAGE gel electrophoresis and stained by Coomassie blue. Prestained protein marker (M) was used, with the first three reference bands having a molecular weight of 250, 150, and 100 kD, respectively. Proteins were excised and identified by MALDI-TOF (see Table S1). The Δ*rpoE* mutant had a reduced expression of the surface antigen I/II SpaP and glucosyltransferase GtfB, while an increased expression of fructan hydrolase FruA can be seen. The lower band was probably truncated glucosyltransferase GtfD, thus marked as GtfD'.

The proteins of the extracellular matrix were extracted and subjected to SDS-PAGE and the differentially expressed proteins were excised, digested with trypsin, and identified by MALDI-TOF (PMF and MS/MS) (Table S1). As shown in Fig. 4, the Δ*rpoE* mutant had a reduced expression of the cell surface antigen I/II SpaP, glucosyltransferases GtfB, and alcohol-acetaldehyde dehydrogenase AdhE; while increased expression of fructan hydrolase FruA was observed, all of which were consistent with our previous microarray data [26]. AdhE is a bifunctional enzyme that is involved in carbon utilization and alcohol metabolism and its reduced expression has also been detected in our previous proteome study (Xue et al., submitted). The increased amount of fructan hydrolase FruA in the Δ*rpoE* mutant could lead to quicker degradation of fructans, which are suggested as extracellular storage polysaccharides of *S. mutans*. FruA has been shown to be necessary for cariogenicity of *S. mutans* [51] and the FruA enzyme of *S. salivarius* reduced the biofilm formation of *S. mutans* [52]. Thus, the increased level of FruA in the Δ*rpoE* mutant might contribute to its inhomogeneous biofilm structure. The glucosyltransferases GtfB is responsible for synthesis of water insoluble glucans [53], therefore the reduced amount of GtfB in the mutant strain explains its decreased extracellular insoluble polysaccharides as shown above (Fig. 3A). The surface antigen SpaP in *S. mutans* has been reported to contribute to biofilm formation in a glucan-binding independent way [8]. Since both SpaP and GtfB displayed reduced expression in the Δ*rpoE* mutant, the altered biofilm matrix structure and surface attachment shown above indicate a biofilm formation mechanism that is independent of SpaP and glucans. Two protein bands were clearly identified as GtfD, however, according to the molecular weight (about 160 kD), the second band could be truncated proteins. Since GtfD synthesizes water soluble glucans [54], the switch of GtfB to GtfD in the Δ*rpoE* mutant could alter the glucan structure in a way that changes the biofilm matrix structure. Similar observations have been reported previously. The mutation of trigger factor (*ropA*) caused reduced expression of the GtfB and GtfD enzymes, but the Δ*ropA* mutant had an increased biofilm formation compared to the wild type [55].

Biofilm formation is important for *S. mutans* to survive inside the host oral cavity and on other tissues, e.g. heart valves. The Δ*rpoE* mutant produced a reduced amount of the extracellular proteins SpaP and GtfB and formed biofilms with a looser extracellular matrix. However, the dendrite-like structure of the Δ*rpoE* mutant biofilm extracellular matrix might allow firm attachment to surfaces. This could benefit its colonization inside the human organism or tissue, where the concentration of sucrose is low compared to the oral cavity and the expression of Gtfs and SpaP should be low to avoid inducing host immune defense mechanisms. Indeed, the *S. mutans* strains isolated from infective endocarditis patients were lacking the intact Gtfs enzymes and had lower sucrose-dependent adhesion [17]. Moreover, mutant strains defective for the surface antigen SpaP resulted in less phagocytosis by human polymorphonuclear leukocytes, thus had a higher survival rate and caused more severe systemic inflammation [56].

### The Δ*rpoE* mutant strongly bound to the human extracellular matrix (ECM) components

**Figure 5:**
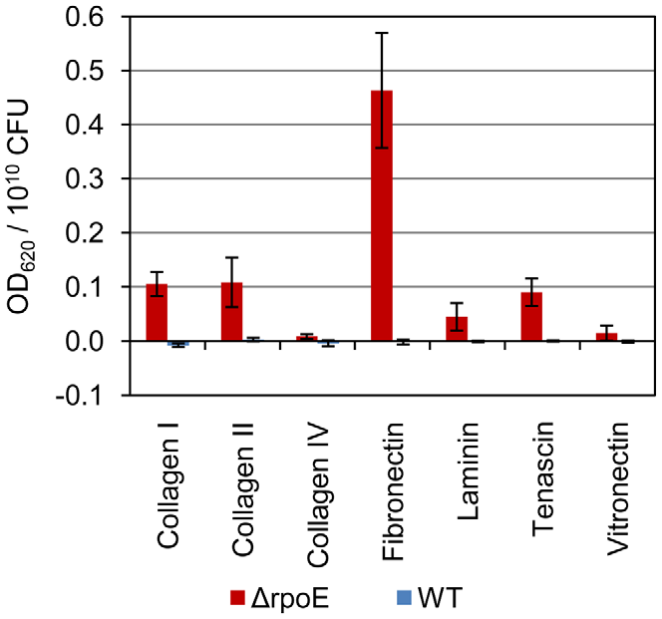
Adherence of *S. mutans* strains to human extracellular matrix (ECM) components. Blue columns: wild type; red columns: Δ*rpoE* mutant. The mean value and standard deviation were calculated from five biological replicates. The Δ*rpoE* mutant adhered more strongly to all tested ECM compounds than the wild type. Binding was especially pronounced for fibronectin.

The ability to bind ECM is one of the major mechanisms for streptococcal pathogenesis [57]. As shown in Fig. 5, the wild type bound poorly to all of the ECM molecules under our experimental conditions. The weak binding of the wild type strain to the ECM components could be due to differences in *S. mutans* strains. Although *S. mutans* has been reported to bind to ECM components, e.g. fibronectin through surface antigen I/II SpaP [58], [59], to the cell wall associated protein WapA [60], [61], the PavA-like protein (SMU. 1449) [2], and AtlA [62], none of these experiments was carried out using the UA159 strain. Moreover, the expression and activity of these receptors is highly regulated by environmental factors [18], thus our experimental conditions could have been not suitable for induction of the high binding activity. By contrast, the Δ*rpoE* mutant effectively bound to the ECM components collagen I, collagen II, tenascin, laminin, and most strongly, to fibronectin. However, the Δ*rpoE* mutant had decreased expression of genes encoding the known ECM binding proteins as reported in *S. mutans*, e.g. surface antigen I/II SpaP, AtlA, and no changed transcription level of WapA and PavA-like protein according to our transcriptome data (microarray GEO record GSE22333). The strong adherence of the Δ*rpoE* mutant to the ECM components, especially fibronectin, must therefore be due to some other differentially expressed surface receptor or modified surface structures.

### The Δ*rpoE* mutant had a reduced adherence to HEp-2 cells

**Figure 6:**
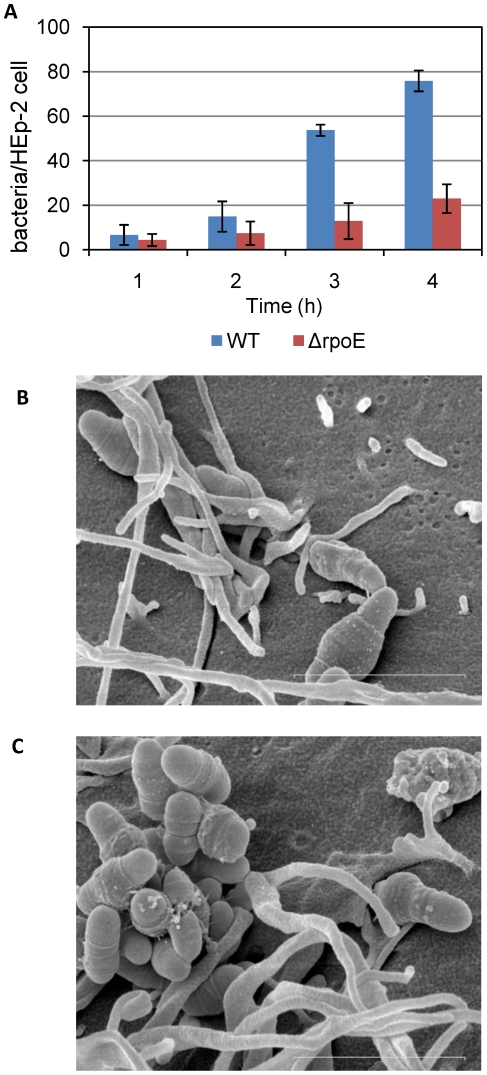
Adherence of *S. mutans* wild type and the Δ*rpoE* mutant to human epithelial cells HEp-2. (A) Quantification of adherent bacteria on HEp-2 cells. The mean value and standard deviation of the ratio of bacteria/HEp-2 cells was calculated from six biological replicates obtained from three independent experiments. More wild type cells adhered to HEp-2 cells. The scanning electron microscope images show the attachment of wild type (B) and the Δ*rpoE* mutant (C) to the surface of human epithelial cells HEp-2. The Δ*rpoE* mutant cells were clumping together. The bars indicate 2 µm.

Because of the strong adherence of the Δ*rpoE* mutant to the ECM components, its adherence to and invasion of host cells was tested using human epithelial cells HEp-2 and primary human large vascular endothelial cells HUVEC. Both the wild type and the Δ*rpoE* mutant showed low adhesion to the endothelial cells HUVEC (data not shown). In comparison, they had much higher adhesion to the epithelial cell line HEp-2 (Fig. 6A). This is consistent with previous reports that *S. mutans* was found to adhere to oral epithelial cells [63], and its biofilm triggered complex host immune responses [64].

Although weakly bound to the ECM matrix, the wild type effectively bound to HEp-2 cells. It may be possible that *S. mutans* adhesion can be triggered by the presence of host cells, or there might be an alternative adhesion mechanism independent of ECM binding. By contrast, the Δ*rpoE* mutant had reduced adherence compared to the wild type, especially at the later time points (after 3 hours of incubation) when both strains started fast multiplication. The scanning electron microscope images show the adhesion of the wild type (Fig. 6B) and the Δ*rpoE* mutant (Fig. 6C) to HEp-2 cells. The Δ*rpoE* mutant tended to clump together when attached to the surface of HEp-2 cells. Thus, the attachment area was relatively small compared to the big mass of the aggregates, and this might have caused easier detachment. Indeed, according to the observation during the experiment, at the later time points, the Δ*rpoE* mutant started to form detached flocs which were easily washed away at the washing step (data not shown).

**Figure 7:**
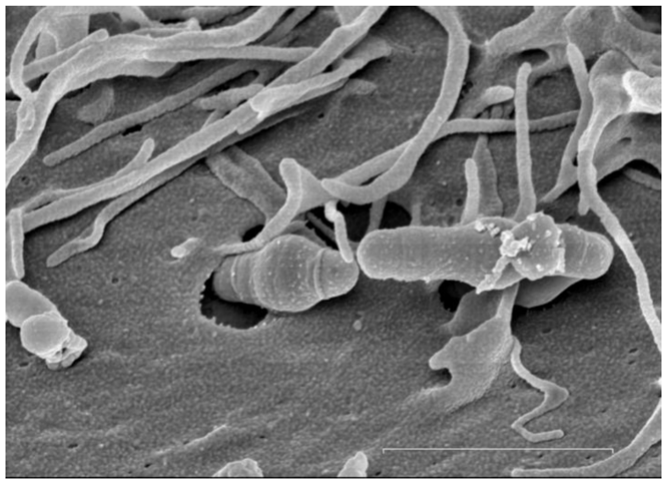
Invasion of human epithelial HEp-2 cells by *S. mutans* wild type cells. The scanning electron microscope image was recorded after 4 hours of incubation of the bacterial culture with HEp-2 cells. The bar indicates 2 µm.

*S. mutans* wild type and the Δ*rpoE* mutant had a low frequency of invasion to both epithelial and endothelial cells (data not shown), indicating that *S. mutans* UA159 derived strains are not strongly invasive. In addition, the lactate dehydrogenase (LDH) enzyme released upon HEp-2 cell lysis was quantified to determine the cytotoxicity upon bacterial adhesion. No obvious differences in the released LDH amount between autolyzed cells and cells to which bacteria adhered were found (data not shown). This suggests that there were no holes in the cell membrane upon *S. mutans* adherence, consistent with the previous finding that serotype *c S. mutans* strains (including strain UA159), although they are the most prevalent strains in dental plaque, are not invasive [15]. However, rare examples of *S. mutans* invading epithelial (Fig. 7) and endothelial cells (Figure S2) could occasionally be observed. It seems that UA159 can invade cells, albeit at a very low level.

The strong adherence of the Δ*rpoE* mutant to the ECM components, especially fibronectin, suggests that it might have a higher potential to bind to host cells. However, probably due to the clumping effect, the Δ*rpoE* mutant could not adhere as well as the wild type on the epithelial HEp-2 cells. Nevertheless, the invasion of human cells could be seen in both the wild type and the Δ*rpoE* mutant occasionally. With its reduced expression of Gtfs and SpaP, the Δ*rpoE* mutant probably has a lower antigenicity. Thus it might survive longer once it invades the host.

### Characterization of the Δ*rpoE* mutant by phenotypic microarray (PM) assays

The PM technology is with 1,920 testable traits the most comprehensive approach for high throughput phenotyping [41]. The system detects the conversion of carbon, nitrogen, phosphate and sulfate sources, but it also monitors the sensitivity for osmotic stress, various heavy metal ions, the pH and inhibitory chemicals. PM assays are performed in microtiter plate format and record the respiration of living cells by the NADH-dependend reduction of a tetrazolium redox dye. The formation of the purple color reflects both the import as well as the metabolic conversion of a specific substrate. The absence of enzymes, e.g. induced by gene knock-outs, results in lack of color formation. Measurement intervals of 15 minutes of the CCD camera are the prerequisite for the generation of PM-kinetics, which provide information about timing and strength of the cell's metabolic activity. The assay is more sensitive than traditional phenotypic growth tests on minimal medium because it also allows to monitor the usage of substrates that are not sufficient for growth [65].

Freshly grown *S. mutans* wild type and mutant cells were inoculated within the complete set of all twenty PM plates (PM 1–PM 20). We established PM data from two biological replicates, e.g. two independent experiments, of both strains in order to investigate the reproducibility of these experiments. Among all twenty plates, the reproducibility was generally high for PM 1, PM 2, and PM 9 to PM 20, as shown in the comprehensive overview in Figure S3. The respiration curves of PM 10 are shown as examples of good reproducibility for both the wild type and the Δ*rpoE* mutant (Figure S3).

The results from PM 3 to PM 8 exhibited a very low reproducibility. The poor metabolic response of *S. mutans* in PM 3 to PM 8 assays has previously been reported [66], [67]. We improved it by modifying the pH of tricarballylic acid (see methods section), but the results were still not satisfactory, thus, the results from PM 3 to PM 8 will not be discussed in this study. Although Biolog PM plates and protocols were conceived to be applicable to diverse bacterial lineages, a further improvement of specific assay conditions, including the provision of supplementary ingredients, is required to obtain optimal results for *S. mutans*.

The PM data from PM 1, 2 and PM 9 to PM 20 were further analyzed by comparing the Δ*rpoE* mutant with the wild type, and a general overview of both biological replicates is shown in the Figure S4. The Δ*rpoE* mutant and the wild type kinetics are colored in green and red, respectively. The predominant occurrence of green signals reflect a conspicuous gain of functions to metabolize sugars (PM 1 & PM 2) and an enhanced resistance against various antibiotics and toxic compounds (PM 9 to PM 20). A complete list of deviating phenotypes of the Δ*rpoE* mutant including the plate type, the well position of each chemical compound and its mode of action is given in the Table S2.

**Figure 8:**
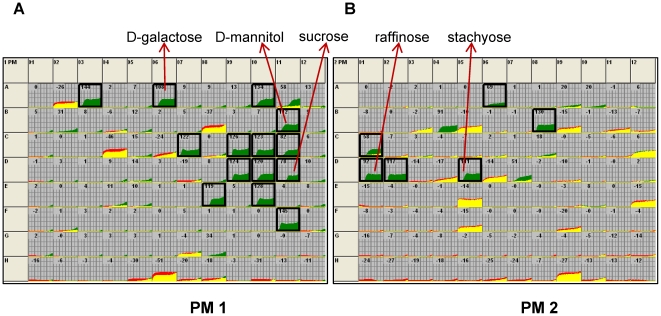
Phenotype Microarray comparison of carbon source utilization by the Δ*rpoE* mutant compared to the wild type. 190 different substrates were tested in the plates PM 1 and PM 2 plates. The metabolic responses of the mutant and the wild type are presented in green and red, respectively, and shared signals are shown in yellow. Black boxes indicate the 20 carbon sources that are exclusively utilized by the mutant, which are reproducible in both experiments. The arrows represent different sugar types that can be metabolized by the Δ*rpoE* mutant, e.g. D-galactose (monosaccharide), sucrose (disaccharide), raffinose (trisaccharide), stachyose (tetrasaccharide), and D-mannitol (sugar alcohol).

The comparison of the Δ*rpoE* mutant with the wild type respiratory activity indicates that the mutant strain metabolized 20 additional carbon sources (Fig. 8). The 20 sugars that were metabolized by the Δ*rpoE* mutant but not by the wild type are highlighted with black boxes, they include mono- (e.g. D-galactose, PM 01, A06), di- (e.g. sucrose, PM 01, D11), tri- (e.g. D-raffinose, PM 01, D 01), and tetra-saccharides (e.g. stachyose, PM 02, D 05) and the sugar derivates (e.g. D-mannitol, PM 01, B 11). The major involved pathways are the galactose and sucrose metabolism (Table 3). *S. mutans* is known to be able to grow on all the carbon sources that were only metabolized by the RpoE mutant in the PM assays. Apparently, the respective enzymes were not synthesized by the wild type under the conditions of the PM assay. The regulatory defect of the RpoE mutant, i.e. its lack of transcriptional specificity, caused it to express a number of enzymes for sugar metabolism which are normally tightly regulated. This is confirmed by our previous transcriptome analysis [26], which suggested an alternative carbon metabolism in the Δ*rpoE* mutant. We showed that the multiple sugar metabolism (MSM) system, which transports and metabolizes various sugars (e.g. raffinose, sucrose, and melibiose) and plays a major role in galactose and sucrose metabolism [3], [68], was highly induced in the Δ*rpoE* mutant [26]. In addition, the upregulation of other genes in the sucrose metabolism pathway, e.g. the sucrose specific transporter (*scrA*) and the sucrose-6-phosphate hydrolase (*scrB*) was also found in our microarray data (microarray GEO data GSE22333). Reproducible weaker metabolic activity of the mutant was only observed on four aldopentoses (PM 1: A02 L-arabinose, B08 D-xylose, C04 D-ribose, H06 L-xylose) that are involved in the pentose phosphate pathway. The difference between wild type and mutant was not above the threshold, but the data are supported by our proteome data showing that the mutant had reduced expression of phosphopentomutase DeoB, which catalyzes the intramolecular transfer of the phosphate group between Ribose-1P and Ribose-5P, an important step in the pentose phosphate pathway (Xue et al., submitted). It would have to be tested if the use of a broader spectrum of carbon sources by the Δ*rpoE* mutant could provide a growth advantage under certain conditions.

**Table 3:** Gained and lost metabolic activity in carbon sources utilization in the *S. mutans* Δ*rpoE* mutant compared to the wild type.

Phenotypes Gained - better metabolic activity				
Plate	Wells^a^	Test	Type of sugar	Pathway involved
PM01	A03	N-Acetyl-D-Glucosamine	Monosaccharide derivative of glucose	Amino sugar and nucleotide sugar metabolism
PM01	A06	D-Galactose	Monosaccharide	Galactose metabolism
PM01	A10	D-Trehalose	Disaccharide	Starch and sucrose metabolism
PM01	B11	D-Mannitol	Sugar alcohol	Fructose and mannose metabolism
PM01	C07	D-Fructose	Monosaccharide	Fructose and mannose metabolism
PM01	C09	α-D-Glucose	Monosaccharide	Glycolysis/Gluconeogenesis
PM01	C10	Maltose	Disaccharide	Starch and sucrose metabolism
PM01	C11	D-Melibiose	Disaccharide	Galactose metabolism
PM01	D09	α-D-Lactose	Disaccharide	Galactose metabolism
PM01	D10	Lactulose	Disaccharide	-
PM01	D11	Sucrose	Disaccharide	Galactose metabolism;Starch and sucrose metabolism
PM01	E08	β-Methyl-D-Glucoside	Monosaccharide derivative of glucose	-
PM01	E10	Maltotriose	Trisaccharide	Starch and sucrose metabolism
PM01	F11	D-Cellobiose	Disaccharide	Starch and sucrose metabolism
PM02	A06	Dextrin	Mixtures of polymers of D-glucose units	Starch and sucrose metabolism
PM02	B08	Arbutin	Glycosylated hydroquinone	Glycolysis/Gluconeogenesis
PM02	C01	Gentiobiose	Disaccharide	-
PM02	D01	D-Raffinose	Trisaccharide	Galactose metabolism
PM02	D02	Salicin	Alcoholic β-glycoside that contains D-glucose	Glycolysis/Gluconeogenesis
PM02	D05	Stachyose	Tetrasaccharide	Galactose metabolism

Phenotypes Lost - less metabolic activity				
Plate Type	Wells	Test	Type of sugar	pathway involved
PM01	H06	L-Lyxose	Aldopentose	Pentose and glucuronate interconversions

a Wells were scored positive if the difference in the height of the metabolic curve was above the threshold value in one experiment and a similar result occurred in the second experiment, albeit sometimes below the threshold value.

The ability of the Δ*rpoE* mutant to maintain respiratory activity in such a large number of assays for chemical sensitivity (PM 9 to PM 20) in contrast to the wild type was unexpected (Table 4, Figure S5). The mutant showed an enhanced resistance against 142 different antibiotics or toxic compounds. For example, metabolic activity of the Δ*rpoE* mutant was observed in the presence of chemicals which affect DNA synthesis, unwinding, and replication. Many inhibitors that block protein synthesis generated positive results for the Δ*rpoE* mutant. The Δ*rpoE* mutant was resistant to many toxic anions and cations, and chemicals that interfere with the tRNA synthetase, cell wall and membrane synthesis. Similar findings were also reported for the mutation of the histidine kinase gene *liaS* in *S. mutans*, which resulted in resistance to antibiotics that are targeting cell-wall biosynthesis, as well as antibiotics inhibiting protein and DNA synthesis [67]. Since antibiotic resistance mechanisms are normally very specific, we speculate that the increased resistance to such a large number of antibiotics could result from general changes in the Δ*rpoE* mutant. The modified surface structure may block the entry of antibiotics and the loosened transcriptional specificity might generate a larger variety of proteins allowing the cell to be readily prepared for facing toxic compounds. An alternative explanation might be the occurrence of secondary mutations in the mutant to compensate for the defects caused by the lack of RpoE. This is a general problem in bacteria. The careful investigation of various phenotypic traits in the Δ*rpoE* mutant and its genetically complemented strain ([26] and unpublished data) did not show indications of secondary mutations. To minimize the possible impact of strain instability, all experiments were carried out with fresh glycerols from the same culture of the mutant.

**Table 4:** Acquired resistance to antibiotics and toxic compounds in the *S. mutans* Δ*rpoE* mutant compared to the wild type.

Mode of Action	Compounds
DNA synthesis	Hexaminecobalt (III) Chloride, Nitrofurantoin, Bleomycin, Trifluoperazine, Myricetin, 5-Fluoro-5′-deoxyuridine, Semicarbazide hydrochloride, Trifluoperazine
DNA intercalator	9-Aminoacridine, 2- Phenylphenol, Coumarin, Umbelliferone
DNA methyltransferase	5-Azacytidine
DNA topoisomerase	Norfloxacin, Ciprofloxacin
DNA unwinding	Oxolinic acid, Pipemidic Acid, Lomefloxacin, Enoxacin, Ofloxacin, Nalidixic acid
folate antagonist	Sulfadiazine, Sulfamethazine, Sulfamethoxazole, Sulfathiazole, Sulfanilamide, Sulfachloropyridazine, Sulfamonomethoxine, Trimethoprim, Sulfisoxazole
Nucleic acid analogs	Azathioprine, 5-Fluorouracil, Cytosine arabinoside
thymidylate synthetase	Trifluorothymidine
ribonucleotide DP reductase	3,5- Diamino-1,2,4-triazole (Guanazole)
protein synthesis	Phenyl-Methyl-Sulfonyl-Fluoride (PMSF), Capreomycin, Spectinomycin, Chloramphenicol, Cinoxacin, Blasticidin S, Rolitetracycline, Tylosin, Oleandomycin, Paromomycin, Tobramycin, Geneticin (G418), Streptomycin, Hygromycin B, Spiramycin, Josamycin, Tetracycline, Amikacin, Gentamicin, Kanamycin, Neomycin, Erythromycin^a^, Lincomycin, Sisomicin, Minocycline
tRNA synthetase	D,L-Serine Hydroxamate, L-Aspartic-b-Hydroxamate, L-Glutamic-g-Hydroxamate
Cell wall synthesis	Glycine, Phosphomycin, Cefoxitin, Cetoperazone, Ampicillin, Moxalactam, Piperacillin, Aztreonam, D-Cycloserine, Cefazolin, Ceftriaxone
toxic anions	Sodium bromated, Sodium periodate, Potassium chromate, Sodium Cyanate, Sodium Arsenate, Sodium Dichromate, Sodium metasilicate, Cobalt chloride, Zinc chloride, Chromium Chloride
toxic cations	Cesium chloride, Nickel chloride, Thallium (I) acetate, Aluminum Sulfate
Toxicity	5-Fluoroorotic Acid, Sodium Nitrate, Sodium Phosphate
membrane	Cetylpyridinium Chloride, Polymyxin B, Colistin
Chelating agents	Sodium pyrophosphate decahydrate, 2,2′-Dipyridyl, 1-Hydroxy-Pyridine-2-thione, Fusaric Acid, 1,10-Phenanthroline
fungicide	Chloroxylenol, Dodine, Nordihydroguaiaretic acid
oxidation	1-Chloro-2,4-Dinitrobenzene, Diamide, Methyl viologen, 3, 4-Dimethoxybenzyl alcohol, Phleomycin
respiration	18-Crown-6-Ether, Sorbic Acid, Pentachlorophenol (PCP), Menadione, Sodium azide, Ruthenium red
Others	X-α-D-Galactoside, X-β-D-Glucoside, X-β-D-Glucuronide, Atropine, Thiosalicylate, Apramycin, Ethionamide, X-PO4, X-SO4, Triclosan, D,L-Propranolol, Caffeine, Aminotriazole, Harmane, D-Serine, Dequalinium, Lidocaine, Tinidazole, 20% Ethylene Glycol, pH 8, Phenylarsine Oxide, b-Chloro-L-Alanine, Trifluoperazine

a *S. mutans* Δ*rpoE* mutant contains an erythromycin resistance gene.

b mode of action includes: α-D-galactosidase, β-D-glucosidase, β-D-glucuronidase, acetylcholine receptor, anti-capsule, antimicrobial, anti-tuberculosic, aryl phosphatase, aryl sulfatase, bacterial fatty acid synthesis, beta-adrenergic blocker, cyclic AMP phosphodiesterase, histidine biosynthesis, imidazoline binding sites, 3PGA dehydrogenase inhibitor, ion channel inhibitor, mutagen, osmotic sensitivity, pH sensitivity, tyrosine phosphatase, aminotransferase inhibitor.

Our previous data showed that the Δ*rpoE* mutant was more sensitive to antibiotics that target protein synthesis, such as tetracycline and kanamycin [26]. However, the PM assays showed that the mutant strain could maintain an active metabolism in contrast to the wild type in the presence of different concentrations of kanamycin (PM 11, H 05–H 08) and tetracycline (PM 12, A 05, 06) (Figure S5). We therefore conducted independent growth tests which showed that the growth conditions strongly influenced the growth and antibiotic resistance of *S. mutans*, and caused these variations. As shown in Figure S6, when grown in 96-well microtiter plates at 37°C without enriched CO_2_ (similar growth condition as in the PM assays), the Δ*rpoE* mutant had growth advantages compared to the wild type in the presence of 100 µg/ml kanamycin, while both strains could overcome the inhibitory effect of 1 µg/ml tetracycline after 48 hours of growth. However, when grown in 96-well microtiter plates at 37°C enriched with 5% CO_2_, the wild type could overcome the inhibitory effect of kanamycin; by contrast, the growth of both strains was inhibited by tetracycline (Figure S6). Our previous antibiotic sensitivity test was carried out at 37°C enriched with 5% CO_2_, but growth was in falcon tubes rather than microtiter plates. As shown in Table S3, under this condition the wild type strain had no difficulty to grow in the presence of both antibiotics, while the Δ*rpoE* mutant failed to grow after the first 20 h, confirming our previous results. Better growth was also found in the closed system with a larger volume of cultures, which left less free space for air than that of smaller volume and thus less oxygen stress occurred. These different cultivation conditions, e.g. the extent of oxygen stress and CO_2_ supplementation, affect the growth and metabolism of both strains in different ways, and thus indirectly result in altered antibiotic sensitivity. In contrast to the drastic effect of growth conditions, the effects of growth media were less pronounced (data not shown). Since antibiotic treatment is still the most important means of disease control [69], the resistance of the Δ*rpoE* mutant to such a large spectrum of antibiotics would make it potentially more difficult to be removed by conventional antibiotic treatment if it were able to colonize a host.

### Conclusion

The results from this study show the multi-dimensional influence of RpoE on virulence related traits of *S. mutans*. The investigated traits are related to plaque formation potentially resulting in damage to host teeth (caries) as well as traits related to adherence, invasion, and survival in host cells and tissues. Both aspects are important for its pathogenesis. Our data are derived from *in vitro* experiments. They do not allow to predict the virulence or competitiveness of the RpoE mutant *in vivo*.

We previously showed that loss of RpoE resulted in massive changes in the transcriptome, and that these changes caused impaired growth, reduced stress tolerance, inhomogeneous biofilm structure, and decreased resistance to tetracycline and kanamycin [26]. The slower growth and weaker resistance to hydrogen peroxide might impair the survival of the mutant in dental plaque, since many of the commensal oral streptococci produce hydrogen peroxide during aerobic growth.In contrast to these findings, we report in this study that the mutant has increased virulence related traits and broader metabolic functions, which however depended strongly on the cultivation conditions. The data show that the transcriptional specificity provided by RpoE restrains the physiology of *S. mutans*. The more or less uncoordinated transcription of a large number of genes in the RpoE mutant results in the synthesis of diverse functional proteins. The work presented here shows that some of the newly expressed traits might increase the virulence of the Δ*rpoE* mutant, e.g. by enhancing its resistance to antibiotics and toxic compounds, reducing its immunogenic surface properties, and altering its carbon metabolism, all of which could result in better survival in the host. Our data also show that many of the virulence related traits essentially depend on cultivation conditions, and thus extrapolation from laboratory data to *in vivo* processes requires extreme caution.

From a genetic perspective, the observed differences of the phenotypes between the *S. mutans* wild type and Δ*rpoE* mutant are striking, especially since their genomes are identical, with the sole exception of the *rpoE* gene replaced by an erythromycin antibiotic resistance gene. The acquired metabolic capacities of the Δ*rpoE* mutant, which occurred simply due to the release of transcriptional specificity, indicate the physiological potential of *S. mutans* UA159. It is conceivable that processes occurring in nature, such as genetic mutations, horizontal gene transfer or environmental changes, could similarly trigger a release of genetic control, thus resulting in comparable phenotypic changes in microbes in the environment. Our data show that we generally see only a fraction of the physiological capabilities of a microorganism by the standard conditions in the laboratory.

## Supporting Information

Supporting Information S1 **PM Procedures for** ***E. faecalis*** **and other Lactic Acid Bacteria.**
(PDF)

Figure S1 **Detachment and inhibition of 16 h old biofilms of** ***S. mutans*** **strains.** (A) Detachment of wild type (WT) and the Δ*rpoE* mutant biofilm by Proteinase K (PK, degrades proteins), DNase I (digests DNA) and NaIO_4_ (oxidizes carbohydrates). (B) Inhibition of *S. mutans* wild type and the Δ*rpoE* mutant biofilm formation by Proteinase K added directly to BMS medium from the beginning of biofilm growth. The biofilms from A and B were quantified by crystal violet staining, and the extracted dye was measured at 620 nm. Mean value and standard deviation were calculated from eight biological replicates from one experiment. The experiment was repeated four times, and the results from one representative experiment are shown here.
(TIF)

Figure S2 **The rare example of invasion of the** ***S. mutans*** **Δ** ***rpoE*** **mutant into human endothelial HUVEC cells.** Samples were fixed after 1 hour of incubation. Images were taken (A) under white light; and (B) using green and red excitation filters to see fluorescent light from antibody marked *S. mutans*, then these two images were merged. Extracellular adherent bacteria were visualized using rabbit polyclonal *S. mutans* antibody (Abcam, USA) and Alexa Fluor 488-conjugated goat anti-rabbit IgG (green). Following permeabilization with 0.01% Triton X-100, extra- and intracellular bacteria were detected by incubation with *S. mutans* antibody followed by an Alexa Fluor 568-conjugated goat anti-rabbit IgG (red). According to their respective label, intracellular bacteria appear red, while extracellular bacteria appear yellow (combined color of green and red). The arrow indicates the intracellular bacteria.
(TIF)

Figure S3 **Reproducibility of Phenotype Microarray (PM) data from two independent experiments.** (A) Overview of PM data from PM 1, 2 and PM 9 to PM 20 (format: upper row left to lowest row right). The curves show respiratory activity determined as formation of a redox dye. The data for the first experiment are shown red, while those for the second experiment are shown green. Thus, perfect reproducibility is indicated by a yellow curve. The upper panel shows the *S. mutans* wild type (WT) and the lower panel shows the Δ*rpoE* mutant in two experiments, respectively. Differences between the two experiments are indicated by red or green colour. The results for PM 1 and PM 2 show good reproducibility for both wild type and mutant. In PM 9 to 20 the wild type showed respiration for some inhibitor compounds which was not observed a second time, indicated by red or green curves. The data for the Δ*rpoE* mutant, which lacked stringent control of gene expression, had a better reproducibility in PM 9 to PM 20 and show that growth was possible for *S. mutans* under these conditions. Thus, the variability seen in the wild type in the two sets of PM experiments could be due to turning on or off certain functional genes by releasing regulatory restraints in response to small changes in cultivation conditions. PM 10 is boxed in red as an example of very good reproducibility for both the wild type and the Δ*rpoE* mutant, which is enlarged in (B). Most results were perfectly reproducible, indicated by yellow curves. Slight changes of the signal strength in the two biological replicates are indicated by red or green margins.
(TIF)

Figure S4 **Overview of Phenotype Microarray (PM) comparison of the** ***S. mutans*** **Δ** ***rpoE*** **mutant with the wild type.** The results from the first and the second experiment are shown in the upper and lower panel, respectively. Yellow indicates similar metabolic activity in both the wild type and the mutant strain. A higher metabolic response of the wild type is indicated in red; while a higher response of the Δ*rpoE* mutant is indicated in green.
(TIF)

Figure S5 **Comparison of the** ***S. mutans*** **Δ** ***rpoE*** **mutant to the wild type in sensitivity assays.** The assays with plates PM 9 to PM 20 were performed in rich medium in the presence of antibiotics or toxic compounds. Yellow indicates similar metabolic activity in both the wild type and the mutant strain. A metabolic advantage by the wild type is indicated in red; while a metabolic advantage by the Δ*rpoE* mutant is indicated in green. The wells with reproducible results in both experiments, and height differences above the threshold in at least one experiment, are highlighted with black boxes. The Δ*rpoE* mutant was more resistant to a large spectrum of or toxic compounds as indicated by many green metabolic curves. The red boxes highlight the resistance of the Δ*rpoE* mutant to 4 different concentrations of kanamycin (PM 11, H 05–H 08) and 2 of the 4 concentrations of tetracycline (PM 12, A 05, A 06).
(TIF)

Figure S6 **Growth of the** ***S. mutans*** **wild type and the Δ** ***rpoE*** **mutant under different conditions.** Bacterial cells were grown in the 96-well microtiter plate at 37°C (A, B) and at 37°C enriched with 5% CO_2_ (C, D). A, C: wild type (WT, solid lines); B, D: Δ*rpoE* mutant (dashed lines). +Tet, +Kan: growth in THBY medium supplied with 1 µg/ml tetracycline (red lines), or 100 µg/ml kanamycin (green lines). The growth of both strains in medium without antibiotics is shown in blue lines.
(TIF)

Table S1 **Identification of the extracellular proteins in the biofilms matrix of** ***S. mutans*** **.**
(DOC)

Table S2 **Gained and lost functions in the** ***S. mutans*** **Δ** ***rpoE*** **mutant compared to the wild type.**
(DOC)

Table S3 **Growth of the** ***S. mutans*** **wild type and the Δ** ***rpoE*** **mutant in falcon tubes.**
(DOC)
