# Supplementary material for: Lack of the Delta Subunit of RNA Polymerase Increases Virulence Related Traits of Streptococcus mutans
Source: PLoS One. 2011 May 19;6(5):e20075. doi: 10.1371/journal.pone.0020075 (PMC3098267; doi:10.1371/journal.pone.0020075)
Supplement: Supporting Information S1 — PM Procedures for E. faecalis and other Lactic Acid Bacteria. (PDF) [file pone.0020075.s001.pdf]

PM Procedures for *E. faecalis* and other Lactic Acid Bacteria

SECTION I: MATERIALS

Section A. List of Equipment, Chemicals and Materials

Table 1. Equipment

Equipment	Source	Catalog #
OmniLog PM System	Biolog	93171, 93182, 93184
Turbidimeter	Biolog	3531, 3532, 3585
Multichannel Pipetter	Biolog	3501A, 3505A and B

Table 2. Chemicals and Materials for Inoculation Procedure

Chemicals and Materials	Source	Catalog #
PM panels 1-20	Biolog	12111, 12112, 12121, 12131, 12141, 12181, 12182, 12183, 12161, 12162, 12211-12220
IF-0a GN/GP Base inoculating fluid (1.2x)	Biolog	72268
IF-10b GN/GP Base inoculating fluid (1.2x)	Biolog	72266
Biolog Redox Dye Mix F (100x)	Biolog	74226
Biolog Redox Dye Mix G (100x)	Biolog	74227
Biolog Redox Dye Mix H (100x)	Biolog	74228
BUG+B agar plates	Biolog	71102
turbidity standard, 85% T	Biolog	3431
sterile cotton swabs	Biolog	3021
sterile pipet tips	Biolog	3001
sterile reservoirs	Biolog	3102
sterile 20 x 150 test tubes	E+K Scientific	266B
sterile 120 ml plastic vial	Capitol Vial Corp.	1-24-786
sealing tape for microplates (optional)	Phenix Research Products	LMT-SEAL-EX
tricarballylic acid	Sigma	T9251
magnesium chloride ($MgCl_2$, $6H_2O$)	Sigma	M0250
calcium chloride ($CaCl_2$, $2H_2O$)	Sigma	C3881
L-arginine, HCl	Sigma	A5131
L-glutamate, Na	Sigma	G1626
L-cystine	Sigma	C8755
uridine-5’-monophosphate (5’-UMP), 2Na	Sigma	U6375
hypoxanthine	Sigma	H9377
β-NAD, hydrate	Sigma	N7004
riboflavin	Sigma	R4500
yeast extract	Oxoid	L21
tween 40	Sigma	P1504
tween 80	Sigma	P1754
D-glucose	Sigma	G8270
pyruvate, Na	Sigma	P2256

Table 3. Composition and Preparation of 12x PM Additive Solutions

Prepare all 120x stock solutions, filter sterilize, and store at 4° C. Combine ingredients and Q.S. to 100 ml. Store at 4° C.

Ingredient	1x Conc.	40-120x Conc.	Formula Weight	Grams/ 100 ml	PM 1,2	PM 3,6,7,8	PM 4	PM 5	PM 9+
tricarballylic acid, pH 5.5 [a]	20mM	800mM	176.1	14.088	-	30ml	30ml	30ml	-
$MgCl_2$, $6H_2O$<br>$CaCl_2$, $2H_2O$	2mM<br>1mM	240mM<br>120mM	203.3<br>147.0	4.88<br>1.76	10ml	10ml	10ml	10ml	10ml
L-glutamate, Na	50uM	6mM	169.1	0.101	10ml	-	10ml	-	-
L-cystine pH8.5 [b]<br>5'-UMP, 2Na	12.5uM<br>25uM	0.5mM<br>1mM	240.3<br>368.1	0.012<br>0.037	30ml	30ml	-	-	-
L-arginine, HCl<br>hypoxanthine<br>β-NAD, hydrate<br>riboflavin<br>yeast extract<br>tween 80 [c]	25uM<br>25uM<br>5uM<br>0.25uM<br>0.005%<br>0.005%	3mM<br>3mM<br>0.6mM<br>30uM<br>0.6%<br>0.6%	210.7<br>136.1<br>663.4<br>376.4<br>-<br>-	0.063<br>0.041<br>0.040<br>0.0011<br>0.6<br>0.6	10ml	10ml	10ml	-	10ml
D-glucose<br>pyruvate, Na [d]	2.5mM<br>5mM	300mM<br>600mM	180.2<br>110.0	5.40<br>6.6	-	10ml	10ml	10ml	10ml
sterile water					40ml	10ml	30ml	50ml	70ml
Total					100ml	100ml	100ml	100ml	100ml

[a]Prepare by adding 14.088g to 55ml of water and then adjust pH to 5.5 with NaOH. Dissolve with stirring and check the pH on the meter and with pH paper. Then Q.S. the final volume to 100ml.

[b]Adjust pH to 8.5 with NaOH and check with pH paper. Check the pH on the meter and with pH paper. Then add the 5'UMP and Q.S. the final volume to 100ml.

[c]Tween 80 is recommended Enterococcus faecalis. Some other species may prefer tween 40.

[d]If the A-1 well in PM3-8 is too false positive, the concentration of the glucose/pyruvate can be decreased to one half or one quarter of this concentration. Alternatively, the pH of the tricarballylic acid buffer can be lowered a little, for example to pH 5.0. If there are no reactions in PM3-8, the pH of the tricarballylic acid buffer can be raised, for example to 6.0, 6.5, or 7.0.

Table 4. Recipe for 1x PM Inoculating Fluids from Stock Solutions

PM Stock Solution	PM1,2 (ml)	PM3,6,7, 8 (ml)	PM4 (ml)	PM5 (ml)	PM9+ (ml)
IF-0a GN/GP (1.2x)	20.0	40.0	10.0	10.0	-
IF-10b GP/GP (1.2x)	-	-	-	-	110.0
Dye mix F, G or H [e] (100x)	0.24	0.48	0.12	0.12	1.32
PM additive (12x)	2.0	4.0	1.0	1.0	11.0
Sterile water	-	-	-	-	-
cells (13.64x)	1.76	3.52	0.88	0.88	9.68
Total	24.0	48.0	12.0	12.0	132.0

[e] Dye mix H is recommended for Enterococcus faecalis. In general, Dye mix F or H is used for fast growing GP genera (Listeria, Enterococcus) and Dye Mix G is used for slow growing GP genera (Streptococcus, Lactococcus, Lactobacillus).

SECTION II: PROCEDURES for PM Inoculation

Section A. Cell Suspension Preparation and PM Inoculation

Preparation of PM Inoculating Fluids

1. Prepare a test tube containing 20 ml of 1x IF-0a.
2. Prepare inoculating fluids as specified in Tables 3 and 4.
3. Dispense inoculating fluids into tubes as diagrammed in Figure 1.

Inoculation of PM Panels (see procedure diagrammed in Figure 1)

Step 1: Prepare Cell Suspension

Grow the bacterium on a BUG+B agar plate by streaking for isolated colonies and allow it to grow overnight at 33 ^{o}C. Subculture a second time.

Remove cells from the BUG+B plate using a sterile swab and transfer into a sterile capped tube containing 20 ml of 1x IF-0a. Stir the cell suspension with the swab to obtain a uniform suspension. Do not vortex or mix turbulently.

Check the turbidity of the suspension; add cells to achieve 81% T (transmittance).

Step 2: Inoculate PM 1,2

Add 1.76 ml of cell suspension to 22.24 ml of PM1,2 inoculating fluid.
Inoculate PM 1 and PM 2 with this cell suspension, 100 ul / well.

Step 3: For PM 3, 6, 7 and 8

Add 3.52 ml of cell suspension to 44.48 ml of PM3,6,7,8 inoculating fluid.
Inoculate PM 3, 6, 7 and 8 with this cell suspension, 100 ul / well.

Step 4: For PM 4

Add 0.88 ml of cell suspension to 11.12 ml of PM4 inoculating fluid.
Inoculate PM 4 with this cell suspension, 100 ul / well.

Step 5: For PM 5

Add 0.88 ml of cell suspension to 11.12 ml of PM5 inoculating fluid.
Inoculate PM 5 with this cell suspension, 100 ul / well.

Step 6: For PM 9+

Add 9.68 ml of cell suspension to 122.32 ml of PM9+ inoculating fluid.
Inoculate PM 9-20 with this cell suspension, 100 ul / well.

Figure 1. PM Procedures for *B. subtilis* and other GP Bacteria

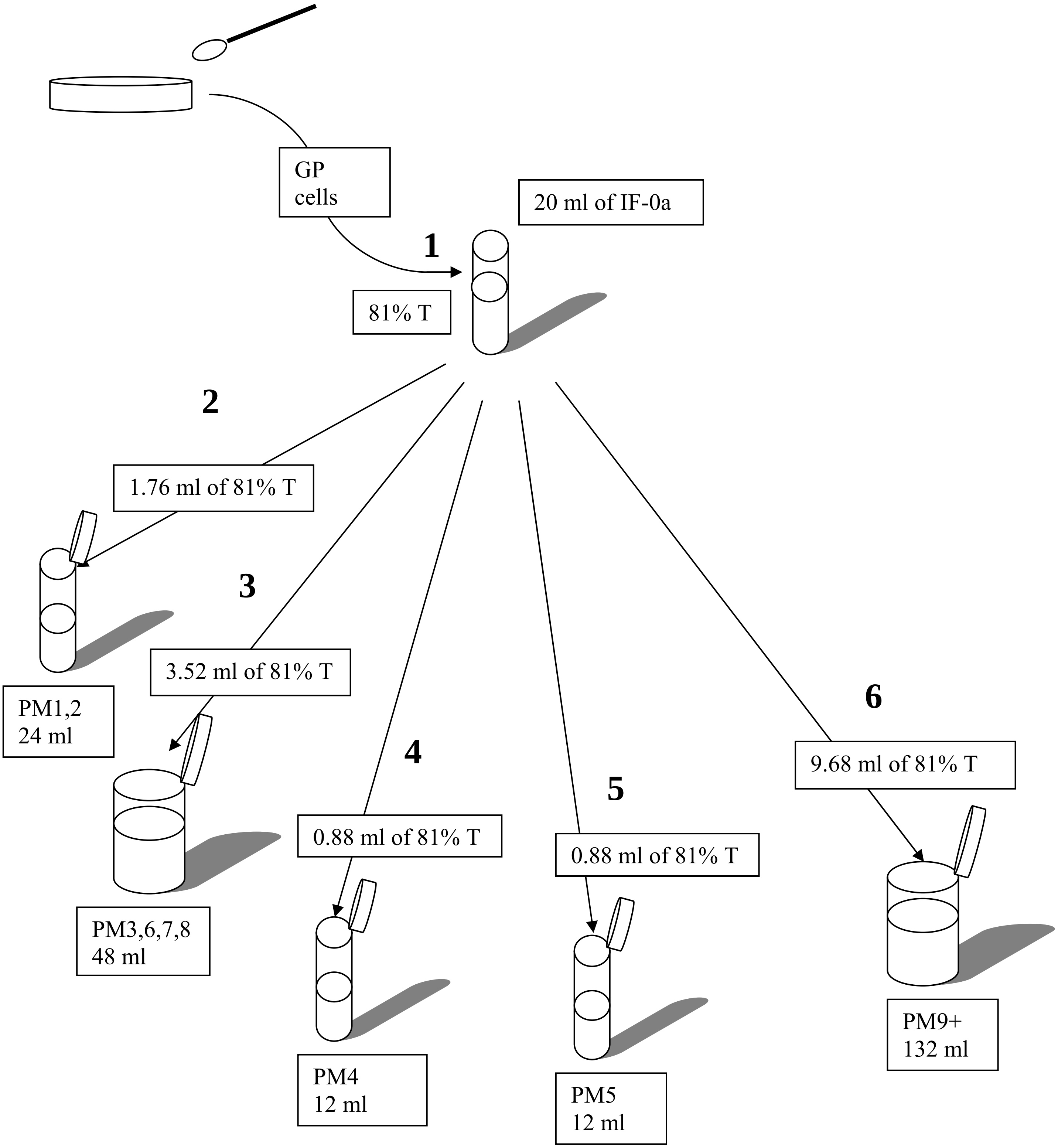

Section B. Incubation and Data Collection

1. Enter worksheet data into OmniLog Software.
2. Load the OmniLog.
3. Incubate all PMs in OmniLog at 30-37°C for 24-48 hours (e.g. 33°C, 36 hr).
4. Remove plates from OmniLog and store at 4°C.
5. Collect the data for analysis.
