# Supplementary figures and images for: Lack of the Delta Subunit of RNA Polymerase Increases Virulence Related Traits of Streptococcus mutans
Source: PLoS One. 2011 May 19;6(5):e20075. doi: 10.1371/journal.pone.0020075 (PMC3098267; doi:10.1371/journal.pone.0020075)

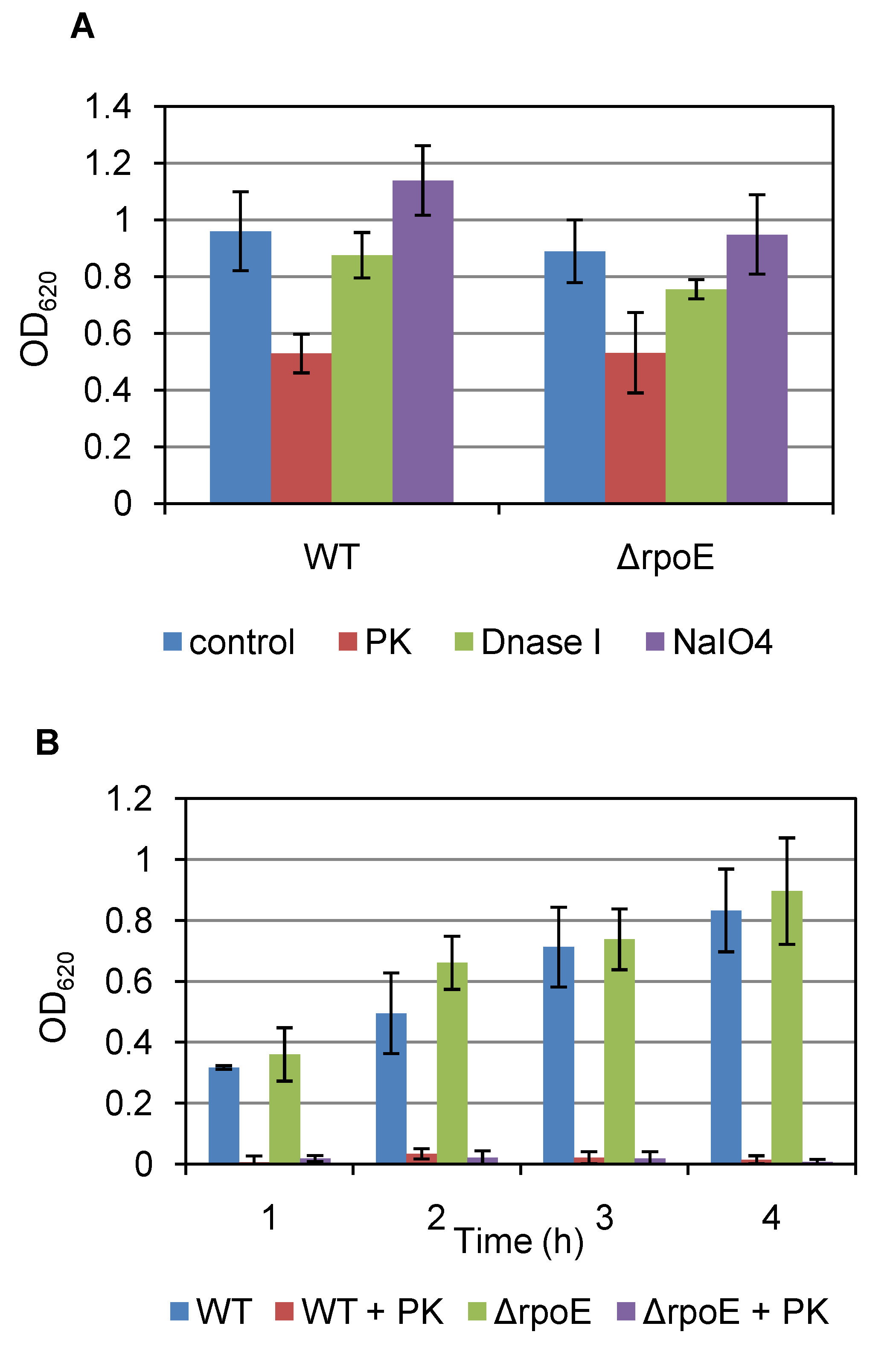

Supplement: Figure S1 — Detachment and inhibition of 16 h old biofilms of S. mutans strains. (A) Detachment of wild type (WT) and the ΔrpoE mutant biofilm by Proteinase K (PK, degrades proteins), DNase I (digests DNA) and NaIO4 (oxidizes carbohydrates). (B) Inhibition of S. mutans wild type and the ΔrpoE mutant biofilm formation by Proteinase K added directly to BMS medium from the beginning of biofilm growth. The biofilms from A and B were quantified by crystal violet staining, and the extracted dye was measured at 620 nm. Mean value and standard deviation were calculated from eight biological replicates from one experiment. The experiment was repeated four times, and the results from one representative experiment are shown here. (TIF) [file pone.0020075.s002.tif]

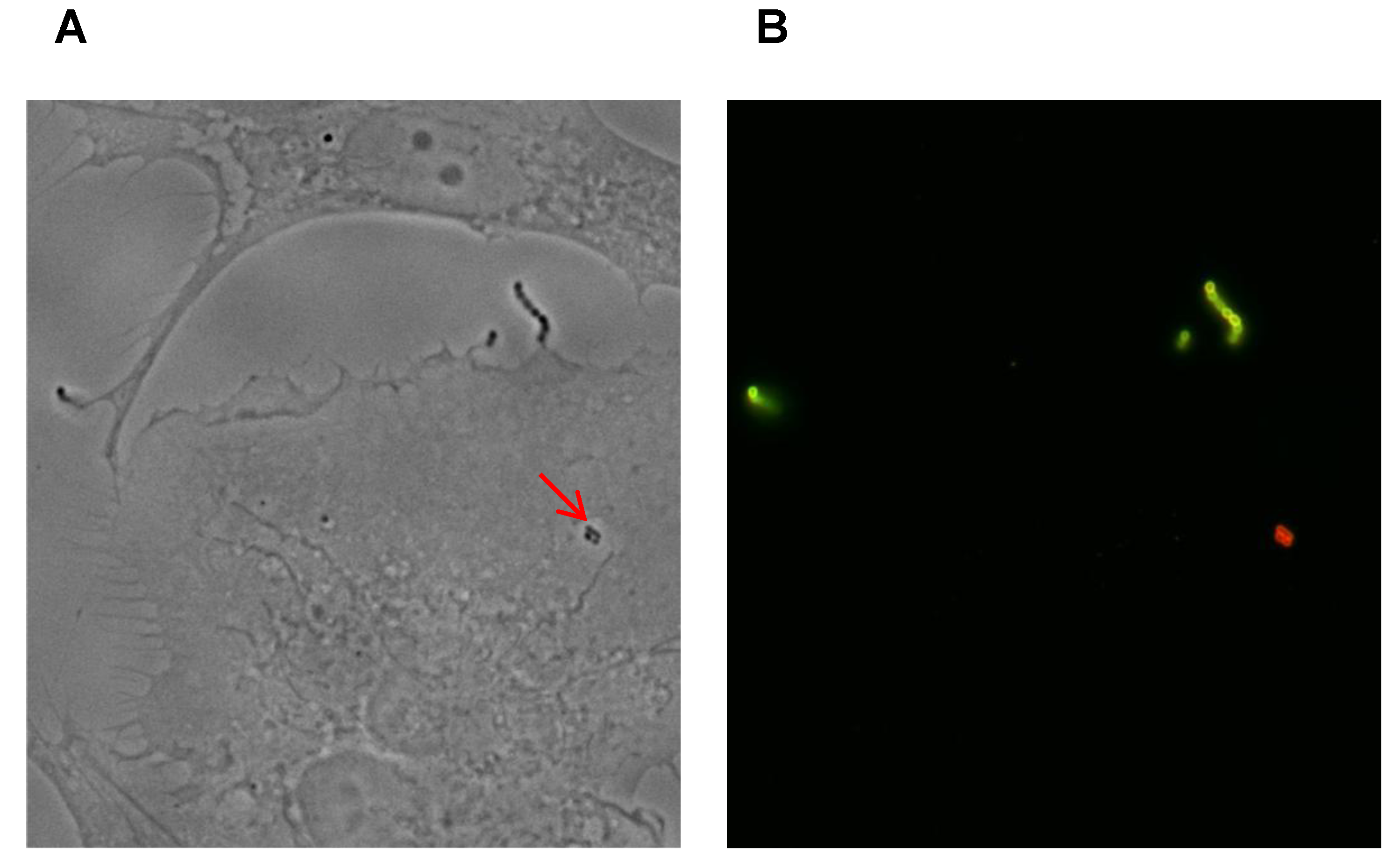

Supplement: Figure S2 — The rare example of invasion of the S. mutans Δ rpoE mutant into human endothelial HUVEC cells. Samples were fixed after 1 hour of incubation. Images were taken (A) under white light; and (B) using green and red excitation filters to see fluorescent light from antibody marked S. mutans, then these two images were merged. Extracellular adherent bacteria were visualized using rabbit polyclonal S. mutans antibody (Abcam, USA) and Alexa Fluor 488-conjugated goat anti-rabbit IgG (green). Following permeabilization with 0.01% Triton X-100, extra- and intracellular bacteria were detected by incubation with S. mutans antibody followed by an Alexa Fluor 568-conjugated goat anti-rabbit IgG (red). According to their respective label, intracellular bacteria appear red, while extracellular bacteria appear yellow (combined color of green and red). The arrow indicates the intracellular bacteria. (TIF) [file pone.0020075.s003.tif]

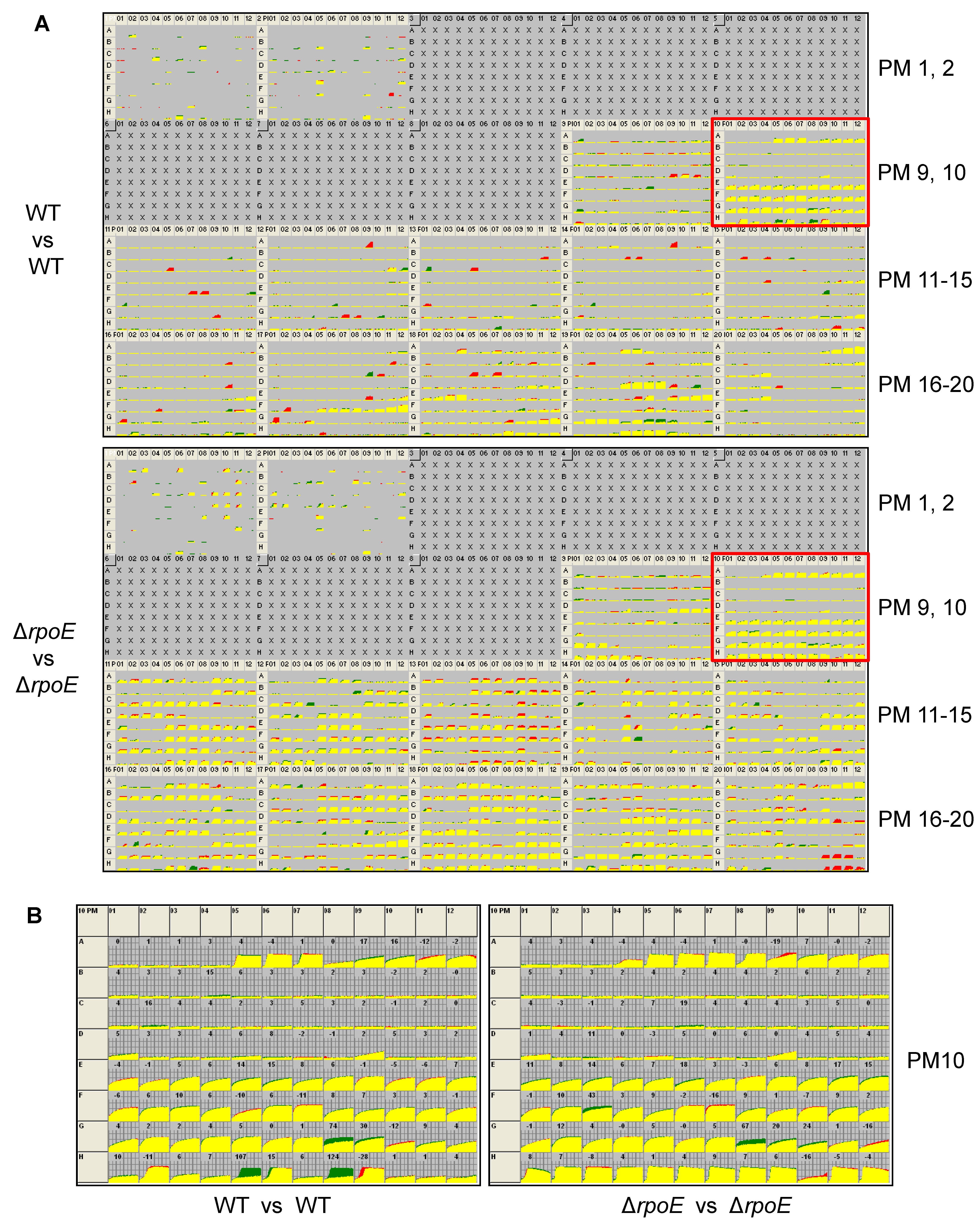

Supplement: Figure S3 — Reproducibility of Phenotype Microarray (PM) data from two independent experiments. (A) Overview of PM data from PM 1, 2 and PM 9 to PM 20 (format: upper row left to lowest row right). The curves show respiratory activity determined as formation of a redox dye. The data for the first experiment are shown red, while those for the second experiment are shown green. Thus, perfect reproducibility is indicated by a yellow curve. The upper panel shows the S. mutans wild type (WT) and the lower panel shows the ΔrpoE mutant in two experiments, respectively. Differences between the two experiments are indicated by red or green colour. The results for PM 1 and PM 2 show good reproducibility for both wild type and mutant. In PM 9 to 20 the wild type showed respiration for some inhibitor compounds which was not observed a second time, indicated by red or green curves. The data for the ΔrpoE mutant, which lacked stringent control of gene expression, had a better reproducibility in PM 9 to PM 20 and show that growth was possible for S. mutans under these conditions. Thus, the variability seen in the wild type in the two sets of PM experiments could be due to turning on or off certain functional genes by releasing regulatory restraints in response to small changes in cultivation conditions. PM 10 is boxed in red as an example of very good reproducibility for both the wild type and the ΔrpoE mutant, which is enlarged in (B). Most results were perfectly reproducible, indicated by yellow curves. Slight changes of the signal strength in the two biological replicates are indicated by red or green margins. (TIF) [file pone.0020075.s004.tif]

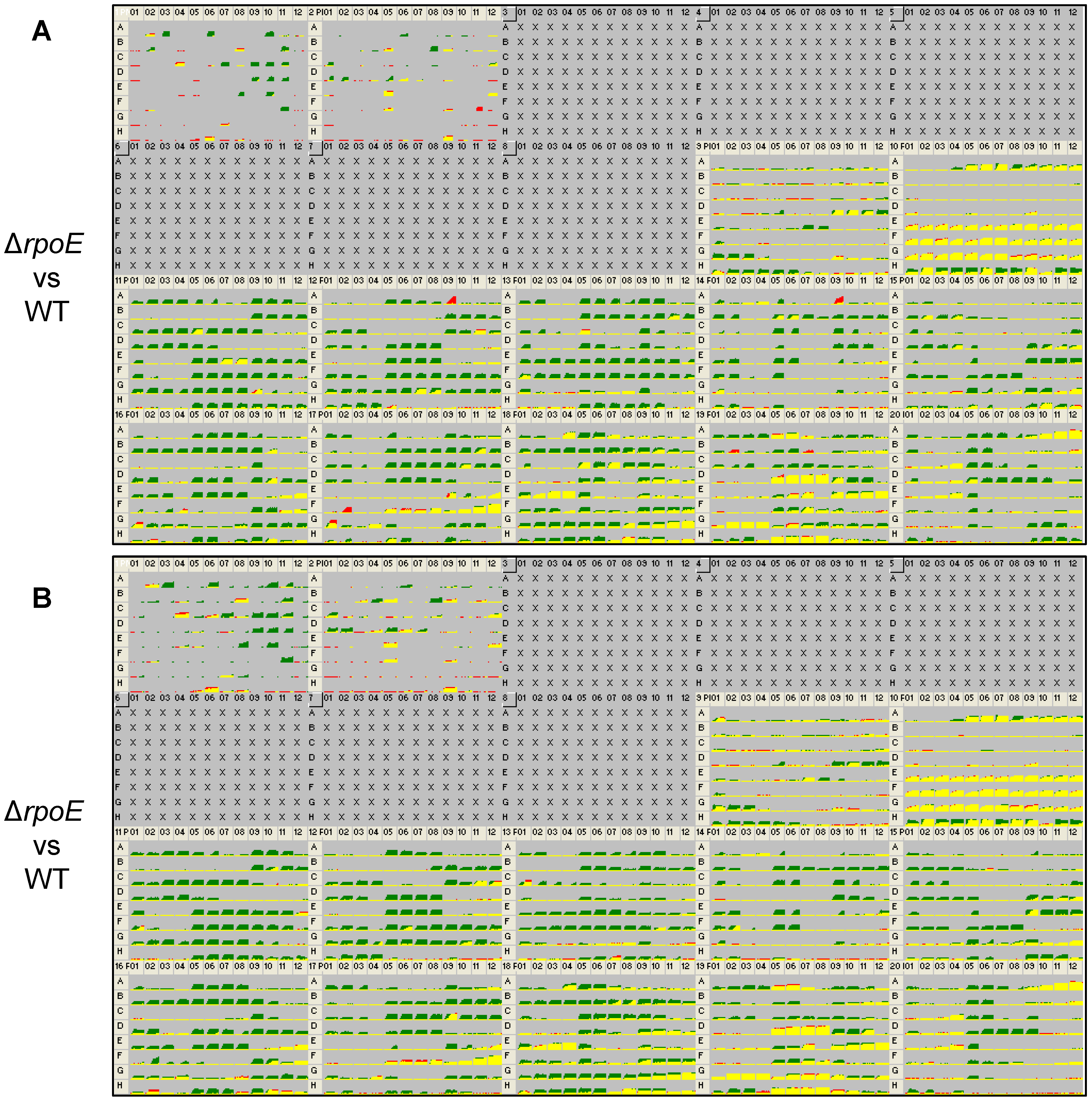

Supplement: Figure S4 — Overview of Phenotype Microarray (PM) comparison of the S. mutans Δ rpoE mutant with the wild type. The results from the first and the second experiment are shown in the upper and lower panel, respectively. Yellow indicates similar metabolic activity in both the wild type and the mutant strain. A higher metabolic response of the wild type is indicated in red; while a higher response of the ΔrpoE mutant is indicated in green. (TIF) [file pone.0020075.s005.tif]

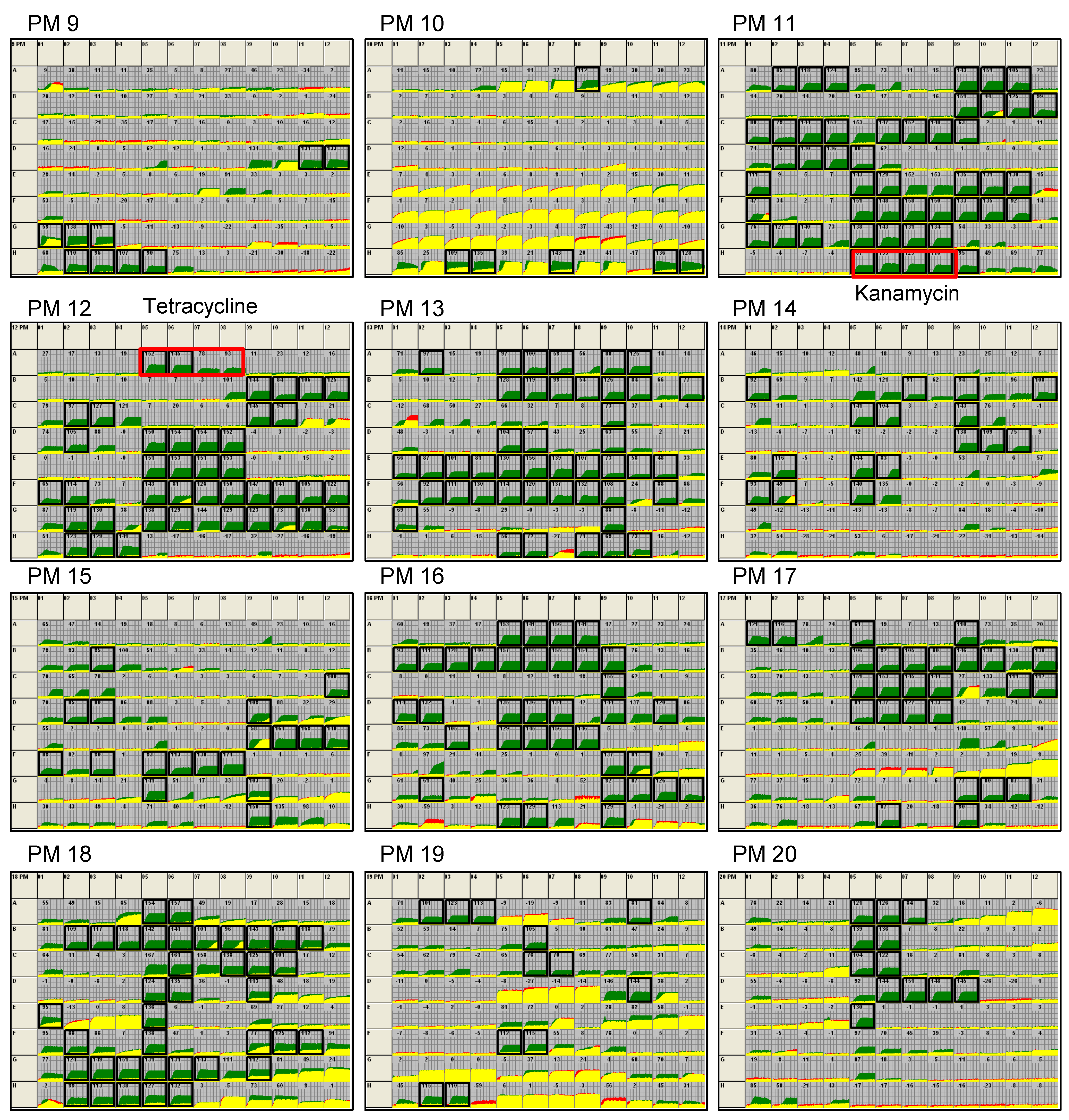

Supplement: Figure S5 — Comparison of the S. mutans Δ rpoE mutant to the wild type in sensitivity assays. The assays with plates PM 9 to PM 20 were performed in rich medium in the presence of antibiotics or toxic compounds. Yellow indicates similar metabolic activity in both the wild type and the mutant strain. A metabolic advantage by the wild type is indicated in red; while a metabolic advantage by the ΔrpoE mutant is indicated in green. The wells with reproducible results in both experiments, and height differences above the threshold in at least one experiment, are highlighted with black boxes. The ΔrpoE mutant was more resistant to a large spectrum of or toxic compounds as indicated by many green metabolic curves. The red boxes highlight the resistance of the ΔrpoE mutant to 4 different concentrations of kanamycin (PM 11, H 05–H 08) and 2 of the 4 concentrations of tetracycline (PM 12, A 05, A 06). (TIF) [file pone.0020075.s006.tif]

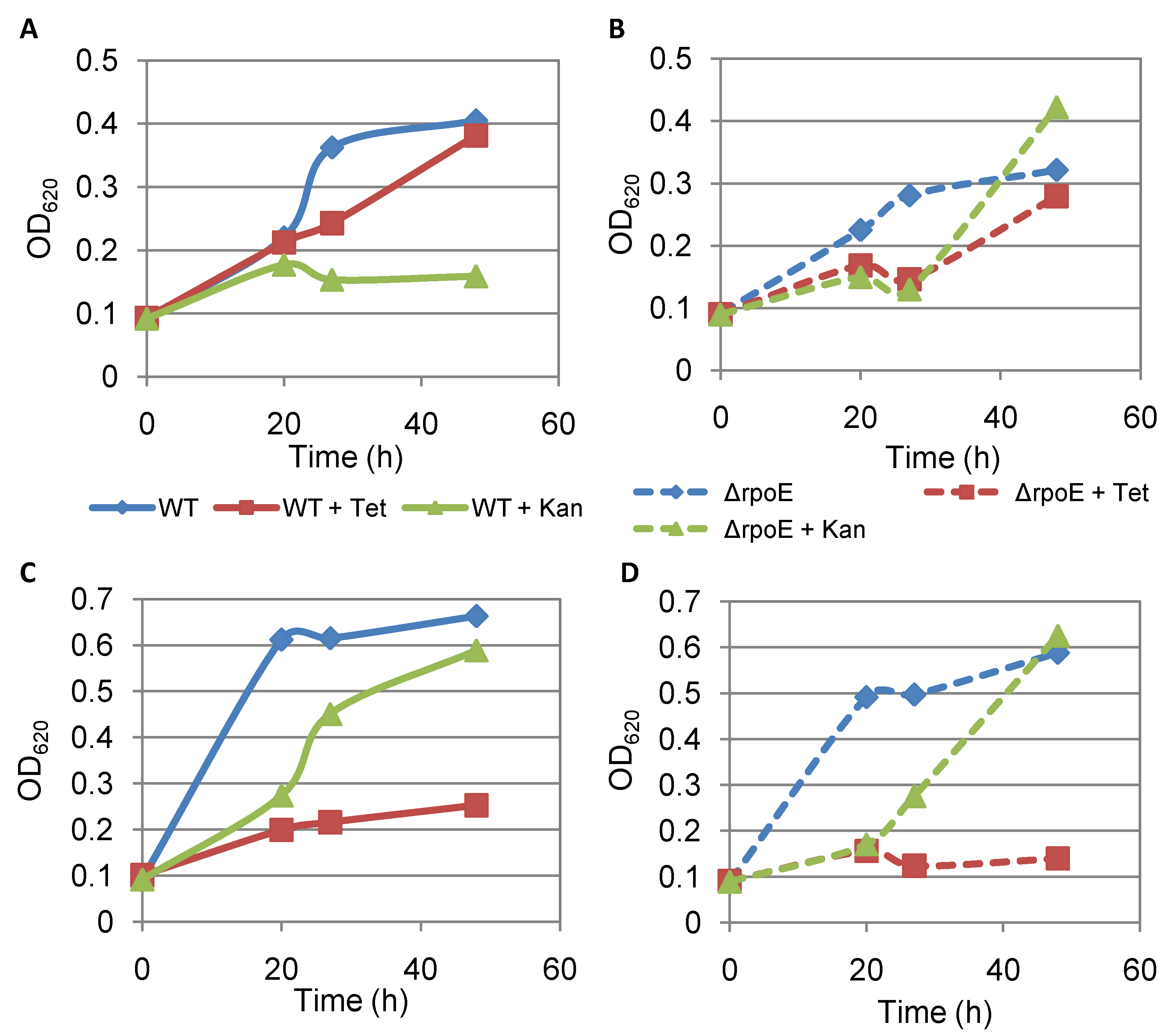

Supplement: Figure S6 — Growth of the S. mutans wild type and the Δ rpoE mutant under different conditions. Bacterial cells were grown in the 96-well microtiter plate at 37°C (A, B) and at 37°C enriched with 5% CO2 (C, D). A, C: wild type (WT, solid lines); B, D: ΔrpoE mutant (dashed lines). +Tet, +Kan: growth in THBY medium supplied with 1 µg/ml tetracycline (red lines), or 100 µg/ml kanamycin (green lines). The growth of both strains in medium without antibiotics is shown in blue lines. (TIF) [file pone.0020075.s007.tif]
