# Supplementary material for: Lack of the Delta Subunit of RNA Polymerase Increases Virulence Related Traits of Streptococcus mutans
Source: PLoS One. 2011 May 19;6(5):e20075. doi: 10.1371/journal.pone.0020075 (PMC3098267; doi:10.1371/journal.pone.0020075)
Supplement: Table S1 — Identification of the extracellular proteins in the biofilms matrix of S. mutans . (DOC) [file pone.0020075.s008.doc]

**Table S1. Identification of the extracellular proteins in the biofilms matrix of *S. mutans*.**

| Protein identification by MALDI-TOF (PMF)^a^ | | | | | | | | | | | |
| --- | --- | --- | --- | --- | --- | --- | --- | --- | --- | --- | --- |
| No.  band | Protein symbol | Gene code | GI No. | | Protein name | Protein MW, Da | Matched peptides | sequence coverage (%) | Protein score | P value | |
| 3 | GtfD' | smu.910 | 153645 | | glucosyltransferase | 159667 | 23 | 18 | 154 | 2.70E-09 | |
| 4 | AdhE | smu.148 | 24378663 | | alcohol-acetaldehyde dehydrogenase | 97331 | 17 | 27 | 142 | 4.30E-08 | |
| 5 | GtfD | smu.910 | 153645 | | glucosyltransferase | 159667 | 20 | 16 | 122 | 4.30E-06 | |
| 6 | FruA | smu.78 | 24378602 | | fructan hydrolase | 158848 | 31 | 28 | 239 | 8.60E-18 | |
| 7 | GtfD' | smu.910 | 153645 | | glucosyltransferase | 159667 | 15 | 12 | 114 | 2.70E-05 | |
| Protein identified by MALDI-TOF/TOF (PMF+ MS/MS)^b^ | | | | | | | | | | | |
| No.  band | Protein symbol | Gene code | GI No. | Protein name | | Protein MW, Da | Peptide for MS/MS | Peptide mass error, Da | Ion  Score | P value | Protein  score |
| 1 | SpaP | smu.610 | 47267 | cell surface antigen I/II | | 170014 | ATAATLATFNADLTK | -0.0755 | 79 | 9.20E-05 | 79 |
| 2 | GtfB | smu.1004 | 153640 | glucosyltransferase-I | | 165594 | NAQGQWFYFDNNGYAVTGAR | -0.1604 | 102 | 1.40E-07 | 99 |
| 2 | GtfB | smu.1004 | 153640 | glucosyltransferase-I | | 165594 | TINGQHLYFR | -0.1124 | 64 | 3.20E-03 | 99 |

^a^Proteins were digested with trypsin and analyzed by a matrix-assisted laser desorption / ionization time of flight mass spectrometry (MALDI-TOF MS) to produce a peptide mass fingerprint (PMF). The obtained peptide masses were used for protein identification using the program MASCOT. Protein score is -10*Log(P), where P is the probability of a random event. Protein scores >81 are significant (P<0.05).

^b^Proteins were digested and analyzed in the same manner as above to produce a peptide mass fingerprint (PMF). The most abundant peptide ions were then subjected to MALDI-TOF/TOF analysis to determine the sequence. The results from both types of analyses were combined and searched for protein identification using the program MASCOT. Ion score is -10*Log(P), where P is the probability of a random event. Ion scores >51 indicate peptide identity or extensive homology (P<0.05). Protein scores are derived from ion scores as a non-probabilistic basis.
