# Supplementary material for: Lack of the Delta Subunit of RNA Polymerase Increases Virulence Related Traits of Streptococcus mutans
Source: PLoS One. 2011 May 19;6(5):e20075. doi: 10.1371/journal.pone.0020075 (PMC3098267; doi:10.1371/journal.pone.0020075)
Supplement: Table S2 — Gained and lost functions in the S. mutans Δ rpoE mutant compared to the wild type. (DOC) [file pone.0020075.s009.doc]

**Table S2. Gained and lost functions in the *S. mutans* Δ*rpoE* mutant compared to the wild type.**

| **Phenotypes Gained - better metabolic activity** | | | |
| --- | --- | --- | --- |
| Plate Type | Wells^a^ | Test | mode of action |
| PM01 | A03 | N-Acetyl-D-Glucosamine | C-source |
| PM01 | A06 | D-Galactose | C-source |
| PM01 | A10 | D-Trehalose | C-source |
| PM01 | B11 | D-Mannitol | C-source |
| PM01 | C07 | D-Fructose | C-source |
| PM01 | C09 | a-D-Glucose | C-source |
| PM01 | C10 | Maltose | C-source |
| PM01 | C11 | D-Melibiose | C-source |
| PM01 | D09 | a-D-Lactose | C-source |
| PM01 | D10 | Lactulose | C-source |
| PM01 | D11 | Sucrose | C-source |
| PM01 | E08 | b-Methyl-D-Glucoside | C-source |
| PM01 | E10 | Maltotriose | C-source |
| PM01 | F11 | D-Cellobiose | C-source |
| PM02 | A06 | Dextrin | C-source |
| PM02 | B08 | Arbutin | C-source |
| PM02 | C01 | Gentiobiose | C-source |
| PM02 | D01 | D-Raffinose | C-source |
| PM02 | D02 | Salicin | C-source |
| PM02 | D05 | Stachyose | C-source |
| PM09 | D11 | 15% Ethylene Glycol | osmotic sensitivity, ethylene glycol |
| PM09 | D12 | 20% Ethylene Glycol | osmotic sensitivity, ethylene glycol |
| PM09 | H02 | 20mM Sodium Nitrate | toxicity, nitrate |
| PM09 | H03 | 40mM Sodium Nitrate | toxicity, nitrate |
| PM09 | H04 | 60mM Sodium Nitrate | toxicity, nitrate |
| PM09 | H05 | 80mM Sodium Nitrate | toxicity, nitrate |
| PM09 | G01 | 20mM Sodium Phosphate pH 7 | toxicity, phosphate |
| PM09 | G02 | 50mM Sodium Phosphate pH 7 | toxicity, phosphate |
| PM09 | G03 | 100mM Sodium Phosphate pH 7 | toxicity, phosphate |
| PM10 | H04 | X-α-D-Galactoside | α-D-galactosidase |
| PM10 | H03 | X-β-D-Glucoside | β-D-glucosidase |
| PM10 | H07 | X-β-D-Glucuronide | β-D-glucuronidase |
| PM10 | H11 | X-PO4 | aryl phosphatase |
| PM10 | H12 | X-SO4 | aryl sulfatase |
| PM10 | A08 | pH 8 | pH, growth at 8 |
| PM11 | C01, C02, C03, C04 | Bleomycin | DNA synthesis; polymerase inhibitor |
| PM11 | B09, B10, B11, B12 | Lomefloxacin | DNA unwinding; gyrase (GN); topoisomerase (GP); fluoroquinolone |
| PM11 | E05, E06 | Enoxacin | DNA unwinding; gyrase (GN); topoisomerase (GP); fluoroquinolone |
| PM11 | H09 | Ofloxacin | DNA unwinding; gyrase (GN); topoisomerase (GP); fluoroquinolone |
| PM11 | E09, E10, E11 | Nalidixic acid | DNA unwinding; gyrase (GN); topoisomerase (GP); quinolone |
| PM11 | C06, C07, C08 | Colistin | membrane; transport |
| PM11 | D02, D03, D04 | Capreomycin | protein synthesis |
| PM11 | F01 | Chloramphenicol | protein synthesis |
| PM11 | A02, A03, A04 | Amikacin | protein synthesis; 30S ribosomal subunit; aminoglycoside |
| PM11 | F09, F10, F11 | Neomycin | protein synthesis; 30S ribosomal subunit; aminoglycoside |
| PM11 | G05, G06, G07, G08 | Gentamicin | protein synthesis; 30S ribosomal subunit; aminoglycoside |
| PM11 | H05, H06, H07, H08 | Kanamycin | protein synthesis; 30S ribosomal subunit; aminoglycoside |
| PM11 | C09 | Minocycline | protein synthesis; 30S ribosomal subunit; tetracycline |
| PM11 | D05 | Demeclocyline | protein synthesis; 30S ribosomal subunit; tetracycline |
| PM11 | F05, F06, F07, F08 | Erythromycin | protein synthesis; 50S ribosomal subunit; macrolide |
| PM11 | A09, A10, A11 | Lincomycin | protein synthesis; lincosamide |
| PM11 | E01 | Cefazolin | wall; cephalosporin first generation |
| PM11 | G01, G02, G03 | Ceftriaxone | wall; cephalosporin third generation |
| PM12 | F09, F10, F11, F12 | 5-Fluoroorotic Acid |  |
| PM12 | D05, D06, D07, D08 | Sulfamethazine | folate antagonist |
| PM12 | E05, E06, E07, E08 | Sulfadiazine | folate antagonist |
| PM12 | F05, F06, F07, F08 | Sulfathiazole | folate antagonist |
| PM12 | G05, G06, G08 | Sulfamethoxazole | folate antagonist |
| PM12 | B09, B10, B11, B12 | Polymyxin B | membrane, outer |
| PM12 | G02, G03 | Spectinomycin | protein synthesis |
| PM12 | C02, C03 | Paromomycin | protein synthesis, aminoglycoside |
| PM12 | D02 | Sisomicin | protein synthesis, aminoglycoside |
| PM12 | F01, F02 | Tobramycin | protein synthesis, aminoglycoside |
| PM12 | H02, H03, H04 | Spiramycin | protein synthesis, macrolide |
| PM12 | A05, A06 | Tetracycline | protein synthesis, tetracycline |
| PM12 | C09, C10 | D,L-Serine Hydroxamate | tRNA synthetase |
| PM12 | G09, G10, G11, G12 | L-Aspartic-b-Hydroxamate | tRNA synthetase |
| PM13 | G09 | Trifluoperazine | cell cycle modulation, DNA synthesis, Ca(2+)/calmodulin dependent protein phosphorylation and lipid |
| PM13 | B05, B06, B07, B08 | 2,2`-Dipyridyl | chelator, Fe++ |
| PM13 | B09, B10, B12 | Oxolinic acid | DNA unwinding, gyrase (GN), topoisomerase (GP), quinolone |
| PM13 | A05, A06, A07 | Dequalinium | ion channal inhibitor, K+ (m) |
| PM13 | D05, D06 | 5-Fluorouracil | nucleic acid analog, pyrimidine |
| PM13 | E01, E02, E03, E04 | Cytosine arabinoside | nucleic acid analog, pyrimidine |
| PM13 | D09 | Rolitetracycline | protein synthesis, 30S ribosomal subunit, tetracycline |
| PM13 | H09, H10 | Tylosin | protein synthesis, 50S ribosomal subunit, macrolide |
| PM13 | E05, E06, E07, E08 | Geneticin (G418) | protein synthesis, aminoglycoside |
| PM13 | E09, E10, E11 | Ruthenium red | respiration, mitochondrial Ca++ porter |
| PM13 | C09 | Potassium chromate | toxic anion |
| PM13 | A09, A10 | Nickel chloride | toxic cation |
| PM13 | F02, F03, F04 | Cesium chloride | toxic cation |
| PM13 | F09, F11 | Thallium (I) acetate | toxic cation |
| PM13 | G01 | Cobalt chloride | toxic cation |
| PM13 | F05, F06, F07, F08 | Glycine | wall |
| PM13 | A02 | Ampicillin | wall, lactam |
| PM13 | H05, H06, H08 | Moxalactam | wall, lactam |
| PM14 | B07 | Fusaric Acid | chelator, lipophilic |
| PM14 | C05, C06 | 1-Hydroxy-Pyridine-2-thione | chelator, lipophilic |
| PM14 | B01 | 9-Aminoacridine | DNA intercalator |
| PM14 | E05, E06 | Nitrofurantoin | DNA synthesis, nitro-compound, multiple sites |
| PM14 | F01, F02 | Chloramphenicol | protein synthesis |
| PM14 | C09 | Sodium Cyanate | transport, toxic anion |
| PM14 | B09, B12 | Sodium Arsenate | transport, toxic anion, PO4 analog |
| PM14 | D09, D10, D11 | Sodium Dichromate | transport, toxic anion, SO4 analog |
| PM14 | E02 | Cefoxitin | wall, cephalosporin |
| PM14 | F05 | Piperacillin | wall, lactam |
| PM15 | C12 | 1,10-Phenanthroline | chelator, Fe++, Zn++, divalent metal ions |
| PM15 | D02, D03 | Phleomycin | DNA damage, oxidative, ionizing ratiation |
| PM15 | D09 | Nordihydroguaiaretic acid | lipoxygenase, fungicide |
| PM15 | E09, E10, E11, E12 | Methyl viologen | oxidizing agent |
| PM15 | F01, F03 | 3, 4-Dimethoxybenzyl alcohol | oxidizing agent, free radical-peroxidase substrate |
| PM15 | F05, F06, F07, F08 | Oleandomycin | protein synthesis, 50S ribosomal subunit, macrolide |
| PM15 | G05 | Sodium azide | respiration, uncoupler |
| PM15 | G09 | Menadione | respiration, uncoupler |
| PM15 | H09 | Zinc chloride | toxic cation |
| PM15 | B03 | D-Cycloserine | wall, sphingolipid synthesis |
| PM16 | E05, E06, E07, E08 | 5-Azacytidine | DNA methyltransferase |
| PM16 | B01, B02, B03, B04 | Norfloxacin | DNA topoisomerase, quinolone |
| PM16 | B05, B06, B07, B08 | Sulfanilamide | folate antagonist |
| PM16 | B09 | Trimethoprim | folate antagonist, dihyldrofolate reductase |
| PM16 | H05, H06 | Chloroxylenol | fungicide |
| PM16 | C09 | Cetylpyridinium Chloride | membrane, detergent, cationic |
| PM16 | D01, D02 | 1-Chloro-2,4-Dinitrobenzene | oxidation, glutathione |
| PM16 | D05, D06, D07 | Diamide | oxidation, glutathione |
| PM16 | D09, D11 | Cinoxacin | protein synthesis |
| PM16 | E03 | Streptomycin | protein synthesis, aminoglycoside |
| PM16 | H09 | Sorbic Acid | respiration, ionophore, H+ |
| PM16 | F09, F10 | Aluminum Sulfate | transport, toxic cation |
| PM16 | G02 | Chromium Chloride | transport, toxic cation |
| PM16 | G09, G10, G11, G12 | L-Glutamic-g-Hydroxamate | tRNA synthetase |
| PM16 | A05, A06, A07, A08 | Phosphomycin | wall |
| PM17 | A05 | b-Chloro-L-Alanine | aa analog, alanine, aminotransferase inhibitor |
| PM17 | A09 | Thiosalicylate | anti-capsule, thiol |
| PM17 | B09, B10, B12 | Ethionamide | anti-tuberculosic |
| PM17 | H06 | Caffeine | cyclic AMP phosphodiesterase |
| PM17 | C05, C06, C07, C08 | Sulfachloropyridazine | folate antagonist |
| PM17 | C11, C12 | Sulfamonomethoxine | folate antagonist |
| PM17 | D05, D06, D07, D08 | Aminotriazole | histidine biosynthesis, catalase |
| PM17 | A01, A02 | D-Serine | inhibits 3PGA dehydrogenase (L-serine and pantothenate synthesis) |
| PM17 | B05, B06, B07, B08 | Hygromycin B | protein synthesis, aminoglycoside |
| PM17 | H09 | Phenylarsine Oxide | tyrosine phosphatase |
| PM17 | G09, G10, G11 | Cetoperazone | wall, cephalosporin |
| PM18 | G02, G03, G04 | Triclosan | bacterial fatty acid synthesis, enoyl-acyl carrier protein reductase |
| PM18 | F02 | Semicarbazide hydrochloride | carbonyl agent, semicarbazide-sensitive amine oxidase, DNA damage |
| PM18 | A05, A06 | Sodium pyrophosphate decahydrate | chelating agent |
| PM18 | G09 | Myricetin | DNA & RNA synthesis, polymerase inhibitor (e. coli) |
| PM18 | H05, H06 | 2- Phenylphenol | DNA intercalator |
| PM18 | B05, B06, B07, B08 | Pipemidic Acid | DNA unwinding, gyrase (GN), topoisomerase (GP), quinolone |
| PM18 | C06, C08 | Sulfisoxazole | folate synthesis, PABA analog |
| PM18 | D09 | Lidocaine | ion channal inhibitor, Na+ |
| PM18 | F05 | Tinidazole | Mutagen, nitroimidazole (GP, GN) |
| PM18 | B09, B10, B11 | Azathioprine | nucleic acid analog, purine |
| PM18 | H02, H03, H04 | 5-Fluoro-5'-deoxyuridine | pyrimidine antimetabolite: inhibits nucleic acid replication |
| PM18 | C09, C10 | Pentachlorophenol (PCP) | respiration, ionophore, H+ |
| PM18 | G05, G06, G07 | 3,5- Diamino-1,2,4-triazole (Guanazole) | ribonucleotide DP reductase |
| PM18 | B02, B03, B04 | Trifluorothymidine | thymidylate synthetase, DNA polymerase |
| PM18 | D05, D06 | Sodium bromate | toxic anion |
| PM18 | E01 | Sodium metasilicate | toxic anion |
| PM18 | E05 | Sodium periodate | toxic anion, oxidizing agent |
| PM18 | F09, F10, F11 | Aztreonam | wall, lactam |
| PM19 | A10 | Coumarin | DNA intercalator |
| PM19 | C06, C07 | Umbelliferone | DNA intercalator |
| PM19 | H02, H03 | Hexaminecobalt (III) Chloride | DNA synthesis |
| PM19 | B06 | Harmane | imidazoline binding sites, agonist |
| PM19 | D10 | Phenyl-Methyl-Sulfonyl-Fluoride (PMSF) | protease inhibitor, serine |
| PM19 | F05, F06 | Blasticidin S | protein synthesis |
| PM19 | A02, A03, A04 | Josamycin | protein synthesis, macrolide |
| PM20 | C05, C06 | Atropine | acetylcholine receptor, antagonist |
| PM20 | A05, A06, A07 | Apramycin | antimicrobial, aminocyclitol |
| PM20 | B05, B06 | D,L-Propranolol | beta-adrenergic blocker |
| PM20 | D06, D07, D08 | Ciprofloxacin | DNA topoisomerase, quinolone |
| PM20 | E05 | Dodine | fungicide, guanidine, membrane permeability |
| PM20 | D09 | 18-Crown-6-Ether | respiration, ionophore |
| **Phenotypes Lost - less metabolic activity** | | | |
| Plate Type | Wells | Test | mode of action |
| PM01 | H06 | L-Lyxose | C-source |

^a^ Wells were scored positive if the difference in the height of the metabolic curve was above the threshold value in one experiment and a similar result occurred in the second experiment, albeit sometimes below the threshold value.
