# Supplementary material for: Lack of the Delta Subunit of RNA Polymerase Increases Virulence Related Traits of Streptococcus mutans
Source: PLoS One. 2011 May 19;6(5):e20075. doi: 10.1371/journal.pone.0020075 (PMC3098267; doi:10.1371/journal.pone.0020075)
Supplement: Table S3 — Growth of the S. mutans wild type and the Δ rpoE mutant in falcon tubes. (DOC) [file pone.0020075.s010.doc]

**Table S3. Growth of the *S. mutans* wild type and the Δ*rpoE* mutant in falcon tubes.**

|  |  | 20 h | | 48 h | |
| --- | --- | --- | --- | --- | --- |
|  |  | 2 ml^b^ | 7 ml | 2 ml | 7 ml |
| WT^a^ | supernatant | +++ | +++ | ++ | +++ |
|  | pellet | + | + | ++ | ++ |
| WT + Tet^c^ | supernatant | + | + | -^d^ | - |
|  | pellet | +++ | ++++ | +++ | ++++ |
| WT + Kan^c^ | supernatant | +++ | +++ | ++ | +++ |
|  | pellet | + | + | ++ | ++ |
| Δ*rpoE* | supernatant | ++ | +++ | + | +++ |
|  | pellet | + | + | ++ | ++ |
| Δ*rpoE* + Tet | supernatant | - | - | - | - |
|  | pellet | - | - | ++++ | ++ |
| ΔrpoE + Kan | supernatant | - | - | + | +++ |
|  | pellet | - | - | ++ | ++ |

^a^ Both wild type (WT) and the Δ*rpoE* mutant initial inoculums were taken from liquid culture at the log growth phase. Bacterial cells were grown at 37°C enriched with 5% CO_2_.

^b^ The volume of inoculums in the 15 ml falcon tubes.

^c^+T, +K: growth in medium supplied with 1 µg/ml tetracycline, or 100 µg/ml kanamycin;

^d^ ‘-’ means no obvious growth, while ‘+’ means slight growth, and with the increase of the number of ‘+’ growth increased.
